# Comparative efficacy of single exercise interventions on pulmonary function and quality of life in patients with chronic obstructive pulmonary disease: a systematic review and network meta-analysis

**DOI:** 10.3389/fmed.2026.1843777

**Published:** 2026-05-28

**Authors:** Xiang Lin, Xueqi Fan, Yanling Huang, Anqi Wang, Yuling Tang, Wen Sun, Ziyan Zhang, Wenshan Xu, Caixia Qiu, Shungui Xu

**Affiliations:** 1 The Affiliated People's Hospital of Fujian University of Traditional Chinese Medicine, Fuzhou, Fujian, China; 2 The Third Affiliated People's Hospital of Fujian University of Traditional Chinese Medicine, Fuzhou, Fujian, China; 3 Fujian University of Traditional Chinese Medicine, Fuzhou, Fujian, China

**Keywords:** chronic obstructive pulmonary disease, Liuzijue, pulmonary function, quality of life, single exercise interventions, Yijinjing

## Abstract

**Objective:**

To systematically compare the effects of various single exercise interventions, including walking, cycling, respiratory muscle training, Taichi, land-based Liuzijue, water-based Liuzijue, Wuqinxi, Baduanjin, Yijinjing, and Yoga, on pulmonary function and quality of life in patients with chronic obstructive pulmonary disease (COPD), and to provide evidence for individualized rehabilitation strategies.

**Methods:**

Randomized controlled trials (RCTs) on single exercise interventions in patients with COPD were systematically searched in PubMed, EMBASE, Web of Science, Cochrane Library, Scopus, and CNKI from database inception to January 12, 2026. The risk of bias of the included studies was assessed using the Cochrane Risk of Bias Tool in RevMan 5.4, and the quality of evidence was further evaluated using the Grading of Recommendations Assessment, Development and Evaluation (GRADE) framework. The network meta-analysis was performed using the “network” command in STATA 17.0.

**Results:**

A total of 77 RCTs involving 5,831 participants were included. The network meta-analysis showed that water-based Liuzijue was the most effective intervention for improving forced expiratory volume in 1 s as a percentage of the predicted value (FEV_1_%pred) (MD = 9.51, 95% CI: 3.23 to 15.78; SUCRA = 88.4%), whereas Yijinjing was the most effective intervention for reducing COPD Assessment Test (CAT) scores (MD = −8.37, 95% CI: −12.83 to −3.91; SUCRA = 95.9%).

**Conclusion:**

Among single exercise interventions for patients with COPD, water-based Liuzijue showed the greatest benefit in improving pulmonary function, while Yijinjing demonstrated the most pronounced effect on quality of life. However, given the limited number of studies on water-based Liuzijue and Yijinjing, these results should be interpreted with caution, and further high-quality randomized controlled trials are needed to validate these findings.

**Systematic review registration:**

https://www.crd.york.ac.uk/PROSPERO/view/CRD420261280662, CRD420261280662.

## 1 Introduction

Chronic obstructive pulmonary disease (COPD) is a common chronic inflammatory respiratory disorder characterized by persistent and often incompletely reversible airflow limitation (1). Its development is closely associated with chronic inflammatory responses in the airways and lung parenchyma triggered by exposure to harmful particles or gases. Clinically, COPD often presents with chronic cough, sputum production, dyspnea, and reduced exercise tolerance, which not only impair pulmonary function but also significantly affect patients’ daily activities and quality of life (2, 3). As a major global public health concern, COPD is associated with high prevalence, morbidity, and mortality, as well as substantial disability and economic burden (4, 5). Epidemiological studies have shown that in 2021, COPD caused approximately 3.5 million deaths worldwide, accounting for about 5% of all deaths globally and ranking as the fourth leading cause of death (6). With population aging and the continued presence of risk factors such as smoking, air pollution, and occupational dust exposure, the global prevalence and burden of COPD are expected to increase further (7, 8). Therefore, exploring safe, effective, scalable, and sustainable intervention strategies is of great clinical importance for slowing disease progression, improving prognosis, and reducing the associated socioeconomic burden.

Beyond pharmacological treatment, exercise intervention has been widely recognized as a key component of pulmonary rehabilitation and is increasingly incorporated into the comprehensive management of patients with COPD (9). Evidence suggests that appropriate exercise interventions can improve pulmonary function to some extent, enhance quality of life, increase exercise tolerance, and relieve dyspnea (10, 11). Although multicomponent exercise programs may offer greater efficacy than single exercise interventions (12), single exercise interventions have advantages in feasibility and scalability. Their relatively simple structure makes them easier for patients to understand and master, reduces implementation difficulty, and may improve adherence and long-term participation. In addition, single exercise interventions are easier to standardize and quantify in terms of training intensity, frequency, duration, and delivery, which facilitates clinical study design, efficacy evaluation, and comparisons across different interventions. They are also more accessible and easier to implement in primary care and community rehabilitation settings, suggesting good potential for real-world application.

In recent years, increasing attention has been paid to single exercise interventions, such as walking, cycling, respiratory muscle training (RMT), Taichi, land-based Liuzijue (LBL), water-based Liuzijue (WBL), Wuqinxi, Baduanjin, Yijinjing, and Yoga, in COPD rehabilitation. However, the effects of these interventions on pulmonary function, quality of life, and exercise capacity remain inconsistent (13–17). Previous network meta-analyses have shown that, among various single exercise interventions, core muscle elastic band training has the most pronounced effect on improving exercise capacity in patients with COPD (18). Nevertheless, systematic comparisons and comprehensive evaluations of the relative efficacy of different single exercise interventions on pulmonary function and quality of life in patients with COPD are still lacking. Therefore, this study used a network meta-analysis to systematically compare the effects of different single exercise interventions on pulmonary function and quality of life in patients with COPD and to rank their relative efficacy, with the aim of providing evidence for individualized rehabilitation strategies in clinical practice.

## 2 Methods

### 2.1 Protocol

This systematic review and network meta-analysis was registered in the International Prospective Register of Systematic Reviews (PROSPERO; registration number: CRD420261280662). The study was conducted in accordance with the Cochrane Handbook for Systematic Reviews of Interventions and reported in accordance with the Preferred Reporting Items for Systematic Reviews and Meta-Analyses extension statement for network meta-analyses (PRISMA-NMA) guidelines (19).

### 2.2 Search strategy

We systematically searched PubMed, EMBASE, Web of Science, Cochrane Library, Scopus, and CNKI from database inception to January 12, 2026. The search strategy used a combination of Medical Subject Headings (MeSH) terms and free-text keywords, including “chronic obstructive pulmonary disease,” “exercise,” “walking,” “cycling,” and “Taichi.” The detailed search strategy is provided in Appendix 1.

### 2.3 Inclusion criteria

In accordance with the PICOS framework, studies were included if they met the following criteria: (1) adult patients (≥18 years) diagnosed with COPD according to the diagnostic criteria of the Global Initiative for Chronic Obstructive Lung Disease (GOLD) guidelines (20); (2) the intervention group received a single exercise intervention, including walking, cycling, RMT, Taichi, LBL, WBL, Baduanjin, Wuqinxi, Yijinjing, or Yoga; (3) the control group received conventional care, no intervention, or placebo; (4) outcomes included at least one of the following: forced expiratory volume in 1 s as a percentage of the predicted value (FEV_1_%pred) and COPD Assessment Test (CAT) score; (5) randomized controlled trials (RCTs) were included.

### 2.4 Exclusion criteria

Studies were excluded if they met any of the following criteria: (1) the intervention group received multi-component or combined exercise interventions; (2) the control group received structured rehabilitation training; (3) non-randomized controlled trials, case reports, study protocols, or animal studies; (4) review articles, conference abstracts, or theses; (5) studies with incomplete data or data that could not be transformed; (6) duplicate publications; and (7) studies for which the full text was unavailable.

### 2.5 Study selection and data extraction

Two reviewers independently performed study selection and data extraction, and the results were cross-checked. Any discrepancies were resolved through discussion, and if consensus could not be reached, a third reviewer was consulted for adjudication. All retrieved records were imported into EndNote X9 for management, and duplicate records were removed. The two reviewers then independently screened the titles and abstracts according to the predefined inclusion and exclusion criteria. Full texts of potentially eligible studies were subsequently obtained and independently assessed to determine final eligibility. Extracted data included the first author, year of publication, country, sample size, participant age, intervention and control measures, intervention frequency and duration, and outcome indicators. For studies reporting data in non-standard formats, the values were converted into endpoint means and standard deviations according to the methods recommended in the Cochrane Handbook for Systematic Reviews of Interventions (21). Detailed procedures are provided in Appendix 2.

### 2.6 Assessment of risk of bias and quality of evidence

The risk of bias of the included studies was assessed using the Cochrane Risk of Bias Tool (22), which covers six domains: random sequence generation, allocation concealment, blinding, completeness of outcome data, selective reporting, and other sources of bias. Each domain was rated as low, high, or unclear risk.

The quality of evidence for each outcome was further assessed using the Grading of Recommendations Assessment, Development and Evaluation (GRADE) approach. Five domains were considered for downgrading, including risk of bias, inconsistency, indirectness, imprecision, and Other Considerations (23). Based on these assessments, the quality of evidence was classified as very low, low, moderate, or high.

### 2.7 Statistical analysis

Pairwise and network meta-analyses were conducted using STATA 17.0, and risk of bias was assessed with RevMan 5.4. For all outcomes, pairwise meta-analyses were first performed, and heterogeneity across studies was evaluated using the I^2^ statistic. A random-effects model was applied when substantial heterogeneity was present (I^2^ ≥ 50%); otherwise, a fixed-effects model was used (24). For the network meta-analysis, a multivariate random-effects model was implemented using the “Network” package in STATA 17.0, which accounts for heterogeneity arising from clinical and other factors across studies and provides more conservative confidence intervals for pooled effect estimates. For continuous outcomes, including FEV_1_%pred and CAT score, endpoint means at the end of the intervention and their corresponding standard deviations were extracted for analysis. Because all studies used the same measurement scales, effect sizes were expressed as mean differences (MDs) with 95% confidence intervals (CIs). Network plots, forest plots, cumulative ranking probability plots, and funnel plots were generated, and relevant statistical parameters were calculated. When closed loops were present, local inconsistency was assessed using the node-splitting method, and loop inconsistency tests were used to examine inconsistency between direct and indirect evidence. The surface under the cumulative ranking curve (SUCRA) was used to rank the relative efficacy of different interventions. SUCRA values range from 0 to 1, with values closer to 1 indicating a greater likelihood of being the best intervention. Sensitivity analyses were performed to assess the robustness of the results. Publication bias and small-study effects were evaluated by visual inspection of funnel plot symmetry and further assessed using Egger’s test.

## 3 Results

### 3.1 Selection process

**Figure 1:**
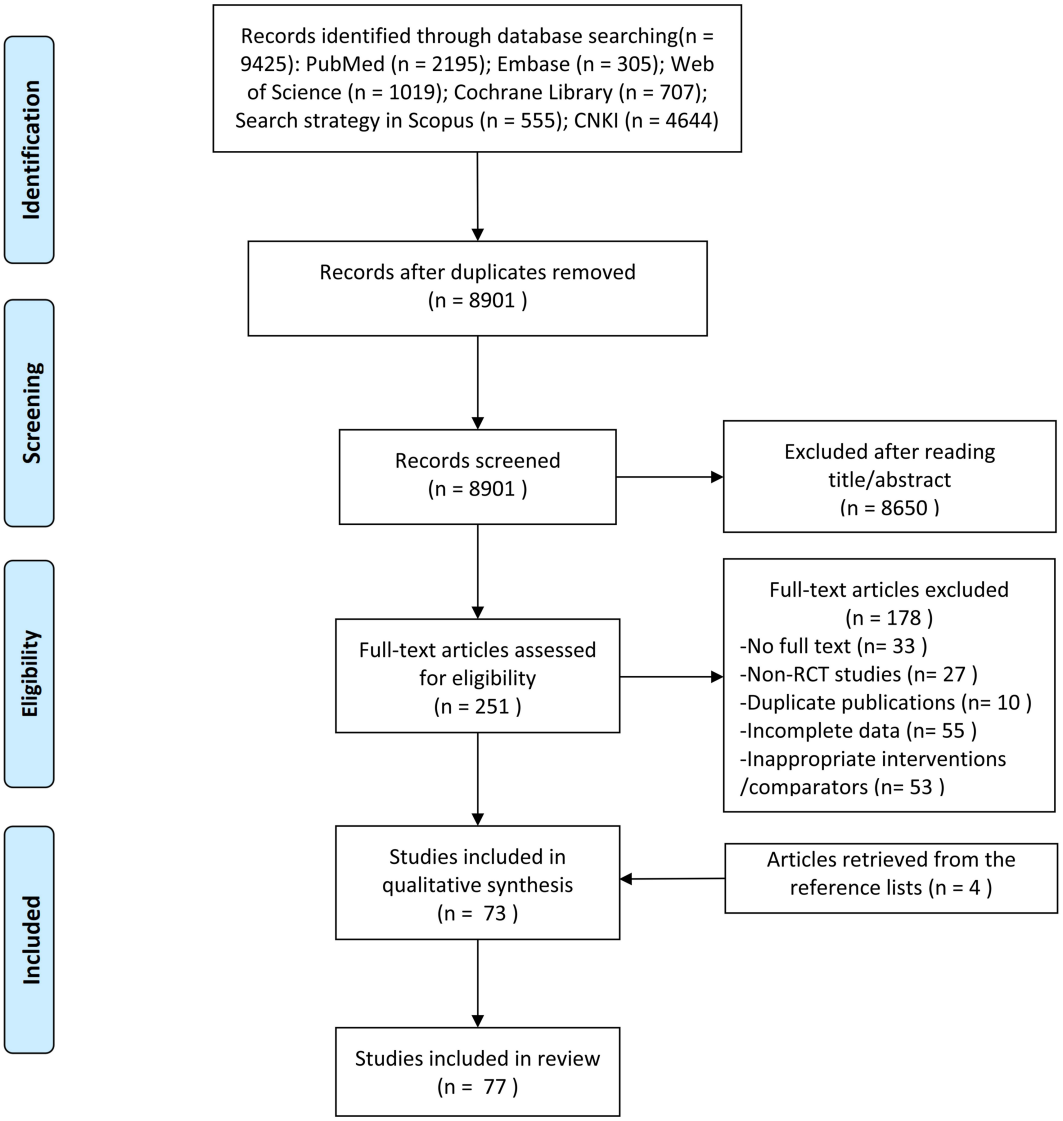
PRISMA flow diagram of the study selection.

According to the predefined search strategy, a total of 9,425 records were initially identified. After duplicate records were removed, the titles and abstracts of the remaining records were screened, and 251 articles were selected for full-text review. Studies that did not meet the inclusion criteria were subsequently excluded, leaving 73 eligible studies. In addition, four studies were identified through manual screening of the references of relevant reviews and original articles. Ultimately, a total of 77 studies were included in the network meta-analysis. The PRISMA-NMA flow diagram of the study selection process is shown in Figure 1.

### 3.2 Characteristics of included studies

A total of 77 studies involving 5,831 participants were included, with 2,988 participants in the intervention groups and 2,843 in the control groups. Among the included studies, the distribution of single exercise interventions was as follows: walking (6 studies), cycling (6 studies), RMT (8 studies), Taichi (9 studies), LBL (12 studies), WBL (3 studies), Wuqinxi (6 studies), Baduanjin (22 studies), Yijinjing (2 studies), and Yoga (5 studies). Details of all included studies are provided in Appendix 3.

### 3.3 Risk of bias assessment and evidence quality evaluation

**Figure 2:**
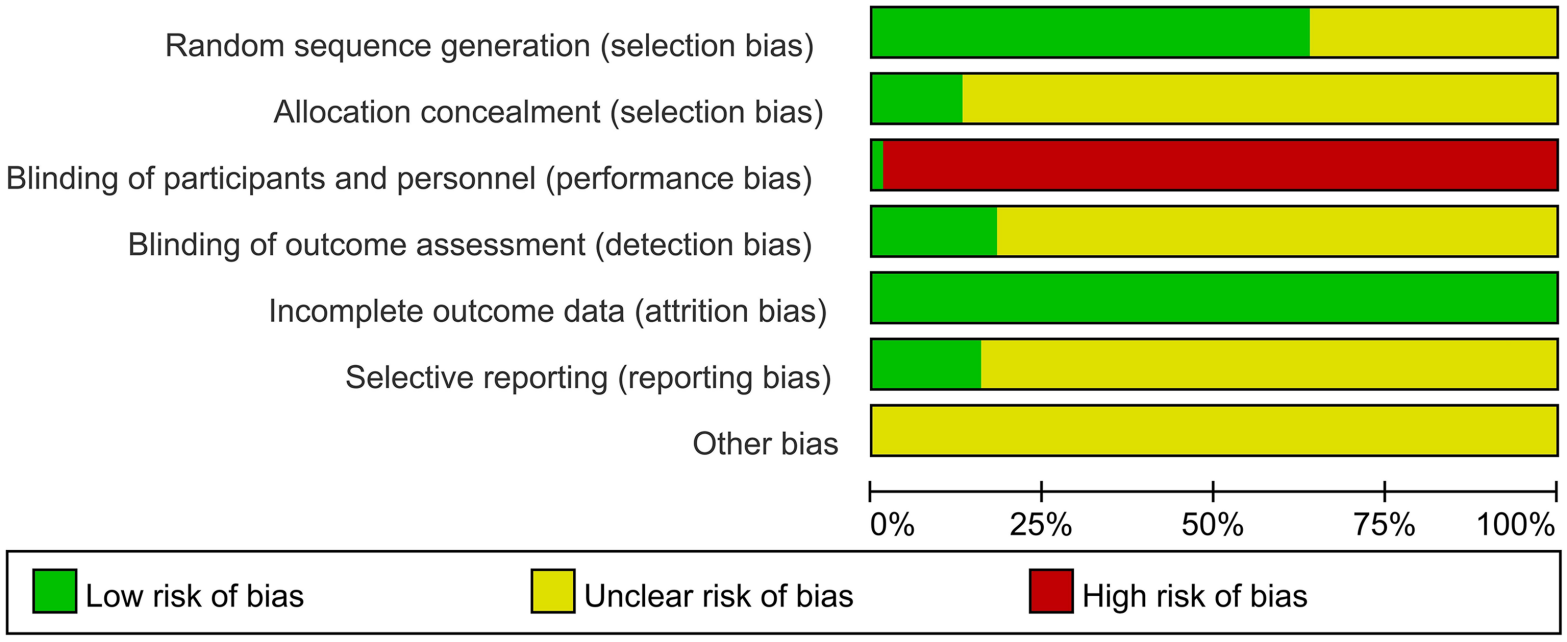
Risk of bias graph.

The results of the risk of bias assessment are shown in Figure 2. Among the 77 included randomized controlled trials, 49 studies reported appropriate methods for random sequence generation, whereas allocation concealment was less frequently described, with only 10 studies explicitly reporting specific procedures. Owing to the nature of the interventions, blinding of participants and personnel was difficult to implement, and this domain was therefore mostly judged as high risk. Fourteen studies reported blinding of outcome assessors. Overall, outcome data were relatively complete, and the risk of selective reporting was low, although assessment was difficult in a small number of studies because of insufficient reporting. Other sources of bias were mostly rated as unclear. Detailed results of the risk of bias assessment for each study are provided in Appendix 4.

Considering the limitations in the implementation of blinding for participants and outcome assessors in the included randomized controlled trials, as well as the relatively small sample sizes and wide confidence intervals, the overall quality of evidence for FEV_1_%pred and CAT was rated as low. Detailed ratings for each outcome are presented in Appendix 5.

### 3.4 Assessment of inconsistency

For the outcomes FEV_1_%pred and CAT score, inconsistency within the network was evaluated using inconsistency tests, node-splitting analysis, and loop-specific inconsistency tests. The results indicated that there were no statistically significant differences between direct and indirect comparisons for either outcome (all *p* > 0.05), suggesting that no substantial inconsistency was detected within the network. Detailed results are provided in Appendix 6.

### 3.5 Network meta-analysis

#### 3.5.1 Network meta-analysis results for the FEV_1_%pred outcome

**Figure 3:**
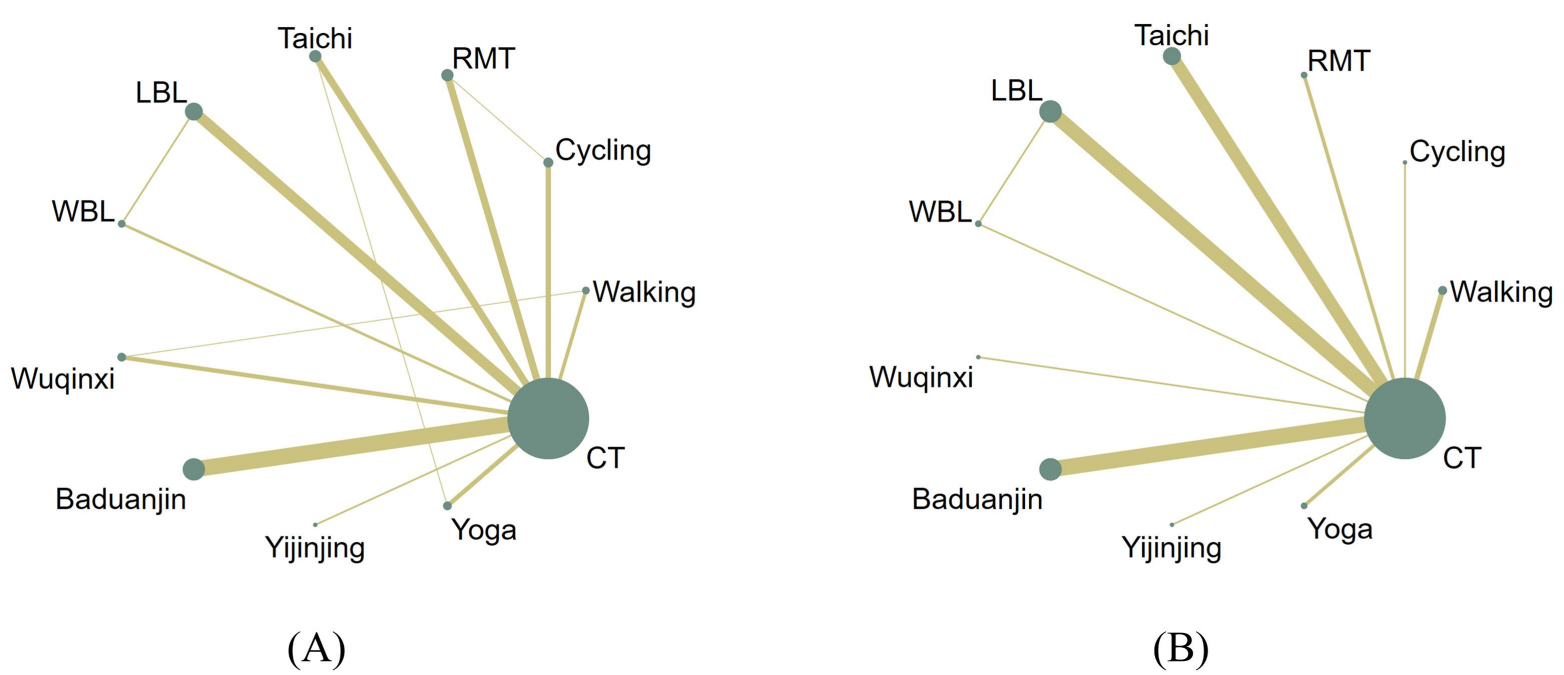
Network evidence plot. **(A)** FEV₁%pred, **(B)** CAT. CT:conventional treatment.

For the FEV_1_%pred outcome, 66 studies involving 4,497 participants were included. The intervention network is shown in Figure 3A. Pairwise meta-analyses were first conducted for interventions with direct comparisons against conventional treatment (CT). The results showed that walking (MD = 2.72, 95% CI: 0.23 to 5.20; I^2^ = 0.0%), LBL (MD = 6.58, 95% CI: 3.17 to 9.45; I^2^ = 71.9%), WBL (MD = 9.72, 95% CI: 5.51 to 13.92; I^2^ = 0.0%), Wuqinxi (MD = 9.57, 95% CI: 2.11 to 17.02; I^2^ = 94.5%), Baduanjin (MD = 5.88, 95% CI: 3.63 to 8.13; I^2^ = 81.8%), Yijinjing (MD = 4.63, 95% CI: 2.10 to 7.16; I^2^ = 51.6%), and Yoga (MD = 4.54, 95% CI: 1.30 to 7.78; I^2^ = 0.0%) were associated with significant improvements in FEV_1_%pred compared with CT, as detailed in Appendix 7.

The network meta-analysis showed that, compared with CT, cycling (MD = 4.76, 95% CI: 0.06 to 9.47), LBL (MD = 6.48, 95% CI: 3.27 to 9.68), WBL (MD = 9.51, 95% CI: 3.23 to 15.78), Wuqinxi (MD = 8.03, 95% CI: 3.68 to 12.39), and Baduanjin (MD = 5.86, 95% CI: 3.54 to 8.19) showed significant advantages in improving FEV_1_%pred. Compared with RMT, LBL (MD = 6.21, 95% CI: 1.09 to 11.33), WBL (MD = 9.24, 95% CI: 1.80 to 16.68), Wuqinxi (MD = 7.77, 95% CI: 1.84 to 13.69), and Baduanjin (MD = 5.60, 95% CI: 0.98 to 10.22) were also significantly more effective. In addition, compared with Taichi, WBL (MD = 7.55, 95% CI: 0.37 to 14.72) and Wuqinxi (MD = 6.07, 95% CI: 0.51 to 11.63) showed significant benefits. Detailed results are provided in Appendix 8.

The SUCRA rankings for FEV_1_%pred improvement were as follows: WBL (88.4%) > Wuqinxi (82.7%) > LBL (70.7%) > Baduanjin (64.8%) > cycling (53.7%) > Yijinjing (52.9%) > Yoga (52.4%) > walking (35.1%) > Taichi (26.9%) > RMT (13.7%) > CT (8.6%), as shown in Table 1 and Appendix 9.

**Table 1:** Ranking table of SUCRA values for FEV_1_%pred and CAT outcomes.

Intervention	FEV_1_%pred	CAT
Walking	35.1	58.7
Cycling	53.7	16.4
RMT	13.7	49.9
Taichi	26.9	77.9
LBL	70.7	33.7
WBL	88.4	60.3
Wuqinxi	82.7	64.4
Baduanjin	64.8	46.4
Yijinjing	52.9	95.9
Yoga	52.4	39.8
CT	8.6	6.6

#### 3.5.2 Network meta-analysis results for the CAT outcome

For the CAT outcome, a total of 34 studies involving 3,121 participants were included. The network of interventions is shown in Figure 3B. First, pairwise meta-analyses were conducted for interventions with direct comparisons against CT. The results indicated that walking (MD = −3.95, 95% CI: −7.21 to −0.68; I^2^ = 88.6%), RMT (MD = −3.33, 95% CI: −6.34 to −0.32; I^2^ = 0.0%), Taichi (MD = −5.16, 95% CI: −7.08 to −3.24; I^2^ = 83.2%), LBL (MD = −2.48, 95% CI: −3.45 to −1.51; I^2^ = 75.6%), and Baduanjin (MD = −3.07, 95% CI: −4.39 to −1.75; I^2^ = 89.9%) all significantly reduced CAT scores, as detailed in Appendix 7.

Network meta-analysis results indicated that compared with CT, walking (MD = −3.86, 95% CI: −6.25 to −1.46), Taichi (MD = −5.06, 95% CI: −6.78 to −3.34), LBL (MD = −2.41, 95% CI: −3.83 to −0.99), WBL (MD = −4.02, 95% CI: −7.62 to −0.42), Wuqinxi (MD = −4.41, 95% CI: −8.57 to −0.25), Baduanjin (MD = −3.08, 95% CI: −4.43 to −1.72), and Yijinjing (MD = −8.37, 95% CI: −12.83 to −3.91) were statistically superior in reducing CAT scores. Moreover, Yijinjing showed significant superiority over cycling (MD = −8.37, 95% CI: −15.57 to −1.17), LBL (MD = −5.96, 95% CI: −10.64 to −1.28), Baduanjin (MD = −5.29, 95% CI: −9.95 to −0.64), and Yoga (MD = −5.78, 95% CI: −11.28 to −0.27); Taichi was also significantly superior to LBL (MD = −2.65, 95% CI: −4.88 to −0.41), as detailed in Appendix 8.

The SUCRA rankings for CAT score reduction indicated the following order of interventions: Yijinjing (95.9%) > Taichi (77.9%) > Wuqinxi (64.4%) > WBL (60.3%) > walking (58.7%) > RMT (49.9%) > Baduanjin (46.4%) > Yoga (39.8%) > LBL (33.7%) > cycling (16.4%) > CT (6.6%), as presented in Table 1 and Appendix 9.

### 3.6 Sensitivity analysis

To assess the robustness of the study results, sensitivity analyses were conducted. Specifically, randomized controlled trials with a sample size smaller than 30 or an intervention duration of less than 12 weeks were excluded to eliminate potential instability or bias introduced by small or short-term studies, and network meta-analyses were re-performed with the remaining studies. The results indicated that the direction of effect, statistical significance, and SUCRA-based ranking of interventions remained largely unchanged compared with the primary analysis. Detailed results are provided in Appendix 10.

### 3.7 Publication bias analysis

**Figure 4:**
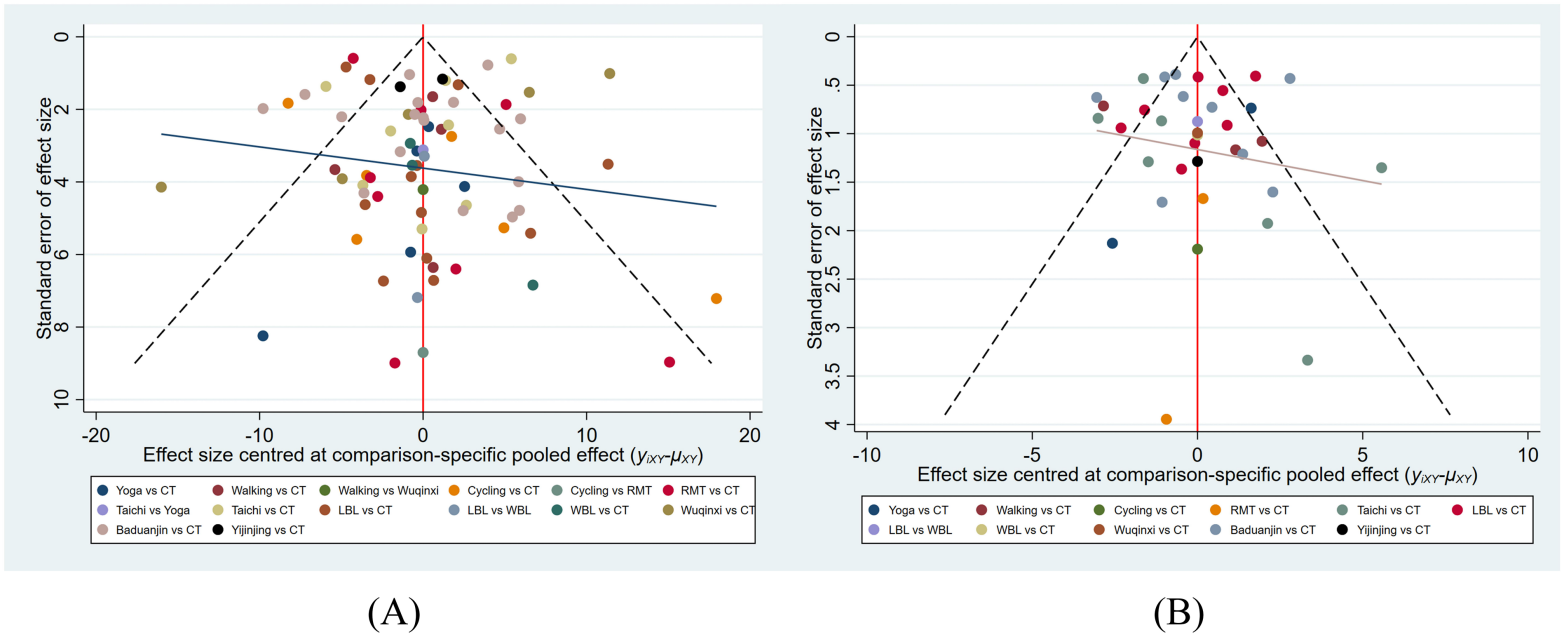
Funnel plots. **(A)** FEV₁%pred, **(B)** CAT. CT:conventional treatment.

Funnel plots were constructed for FEV_1_%pred and CAT outcomes to evaluate publication bias, as shown in Figure 4. Overall, the scatter points in the plots were generally symmetrical; although slight asymmetry was observed, most points were distributed around the central line, indicating no substantial publication bias or small-study effects. The results of Egger’s test further supported these findings, suggesting that no significant publication bias was detected for either the FEV_1_%pred (*p* = 0.316) or CAT (*p* = 0.578) outcomes. Detailed results are provided in Appendix 11.

## 4 Discussion

COPD is a preventable and treatable chronic respiratory disease (25). The American College of Chest Physicians (ACCP) and the American Association of Cardiovascular and Pulmonary Rehabilitation (AACVPR) jointly published evidence-based clinical practice guidelines for pulmonary rehabilitation, identifying exercise training as a core component. Properly implemented pulmonary rehabilitation can significantly improve dyspnea, exercise tolerance, and health-related quality of life in patients with COPD (26). In the present study, the clinical efficacy of different single exercise interventions in patients with COPD was systematically evaluated using a network meta-analysis. A total of 77 randomized controlled trials involving 5,831 participants were included. The main outcomes were FEV_1_%pred and CAT score. The effects of walking, cycling, RMT, Taichi, LBL, WBL, Wuqinxi, Baduanjin, Yijinjing, and Yoga were compared. According to the probability rankings, WBL showed the greatest benefit in improving pulmonary function, whereas Yijinjing had the most pronounced effect on quality of life. These findings suggest that different single exercise interventions may have distinct advantages in COPD rehabilitation.

FEV_1_%pred is an important pulmonary function parameter for assessing the severity of airflow limitation in patients with COPD and is widely used for disease staging and prognosis (27). In the present study, WBL ranked highest for improving FEV_1_%pred, suggesting a potential advantage in improving pulmonary function in patients with COPD. Liuzijue is a traditional exercise that combines breathing training, vocalization, and body movements. It involves six specific vocalizations—“Xu, He, Hu, Si, Chui, Xi”—coordinated with breathing rhythm and corresponding movements, and may help relieve dyspnea, improve pulmonary function, and enhance respiratory muscle function in patients with COPD (28). Some studies have suggested that Liuzijue incorporates features of pursed-lip breathing and reverse abdominal breathing, which may strengthen the respiratory muscles and diaphragm, increase thoracic mobility, improve alveolar ventilation, and regulate breathing patterns. These effects may help prevent premature small airway collapse, improve ventilation, and slow the decline in lung function in patients with COPD (29). WBL is a rehabilitative form of Liuzijue performed in an aquatic environment, combining traditional exercise with hydrotherapy. Hydrotherapy mainly relies on the physical properties of water, including temperature, buoyancy, resistance, and hydrostatic pressure, to support treatment and rehabilitation (30). Compared with land-based exercise, aquatic training can reduce mechanical load through buoyancy, thereby decreasing joint stress and the risk of falls, while water resistance may enhance muscle training. In addition, hydrostatic pressure may have beneficial effects on the thorax and circulatory system, thereby contributing to improvements in respiratory and circulatory function (31, 32). For patients with COPD, this form of training may provide a relatively safe and comfortable exercise environment and may further enhance the effects of breathing training through the influence of the aquatic environment on respiratory patterns (33). Therefore, the beneficial effect of WBL on pulmonary function may be related to the combined effects of breathing training and the supportive aquatic environment. However, the number of available studies on WBL in patients with COPD remains limited, and the current evidence base is still relatively small. To our knowledge, this study is the first to evaluate the effect of WBL on FEV_1_%pred using both pairwise meta-analysis and network meta-analysis. Although the findings appear promising, its effect stability, long-term efficacy, and clinical applicability still need to be confirmed by larger, high-quality randomized controlled trials.

The CAT is an important subjective measure for evaluating health status impairment in patients with COPD. It reflects symptom burden, limitations in daily activities, and the overall impact of the disease on quality of life, making it a useful tool for clinical assessment and follow-up (34). The results of this study showed that Yijinjing had the greatest effect on improving CAT scores, suggesting a potential advantage in improving quality of life in patients with COPD. Yijinjing is a traditional exercise that emphasizes posture regulation, breathing coordination, and mind–body integration. It involves stretching, flexion and extension movements, and postural transitions performed in combination with controlled breathing, which may help improve overall physical function and enhance coordination between breathing and movement (35). For patients with COPD, Yijinjing may not only reduce symptom burden by improving physical activity capacity and respiratory regulation, but also promote psychological well-being through mind–body coordination, thereby contributing to improvements in quality of life. Previous studies have shown that Yijinjing may improve mental health, enhance positive emotional regulation, and help relieve stress, loneliness, and other negative emotions (36). Because CAT scores reflect not only respiratory symptoms, but are also influenced by activity tolerance, sleep quality, and psychological status, the improvement in CAT scores associated with Yijinjing may be related not only to its effects on pulmonary function and physical capacity, but also to its potential benefits in emotional regulation and psychological well-being. In addition, studies have suggested that Yijinjing may have broader health benefits, including improving suboptimal health status, reducing pain, promoting recovery of lumbar function, enhancing immune function, improving sleep quality, and alleviating depressive symptoms (37).

This study systematically searched multiple databases to include relevant studies as comprehensively as possible, without imposing strict restrictions on language. As a result, both English and non-English studies were included, which may have enhanced the comprehensiveness of the evidence. However, several limitations should be considered when interpreting the findings. First, although 77 randomized controlled trials were included with a relatively large overall sample size, the number of studies for different interventions was uneven. In particular, the number of studies on WBL, Yijinjing, and Yoga was relatively small. At the same time, this study included only FEV_1_%pred and CAT scores as outcome measures, which may affect the stability of the effect estimates and the reliability of the intervention ranking results. Second, indirect comparisons in network meta-analysis generally have lower statistical power than direct comparisons, and their results depend on indirect links established through common control groups. Therefore, sparse network structures and heterogeneity may lead to wider confidence intervals and greater uncertainty (38). Third, although most included studies mentioned random allocation, only a few explicitly reported allocation concealment. In addition, because of the nature of exercise interventions, blinding of participants and investigators was often not feasible, which may have increased the risk of performance and detection bias. Finally, there were differences across studies in intervention frequency, duration, training intensity, and conventional treatment, and the substantial heterogeneity may also have influenced the pooled results. In addition, the included single exercise interventions are not a homogeneous category, as they differ fundamentally in mechanisms and characteristics, and such heterogeneity may affect the core transitivity assumption of the network meta-analysis. Therefore, when interpreting SUCRA rankings and the relative effects of interventions, caution should be exercised, taking the specific clinical context into account. Future studies should focus on rigorously designed randomized controlled trials with adequate sample sizes and longer follow-up to further evaluate the efficacy and long-term applicability of different single exercise interventions, thereby providing stronger evidence to optimize individualized rehabilitation strategies for patients with COPD.

## 5 Conclusion

Among single exercise interventions for patients with COPD, WBL showed the greatest benefit in improving pulmonary function, while Yijinjing demonstrated the most pronounced effect on quality of life. However, given the limited number of studies on WBL and Yijinjing, these results should be interpreted with caution, and further high-quality randomized controlled trials are needed to validate these findings.

## Data Availability

The datasets presented in this study can be found in online repositories. The names of the repository/repositories and accession number(s) can be found in the article/Supplementary material.

## References

[1] Agustí A Celli BR Criner GJ Halpin D Anzueto A Barnes P . Global initiative for chronic obstructive lung disease 2023 report: GOLD executive summary. Eur Respir J. (2023) 61:2300239. doi: 10.1183/13993003.00239-2023, 36858443 PMC10066569

[2] Hanania NA O'Donnell DE. Activity-related dyspnea in chronic obstructive pulmonary disease: physical and psychological consequences, unmet needs, and future directions. Int J Chron Obstruct Pulmon Dis. (2019) 14:1127–38. doi: 10.2147/COPD.S188141, 31213793 PMC6538882

[3] Wang Q Liu S. The effects and pathogenesis of PM2.5 and its components on chronic obstructive pulmonary disease. Int J Chron Obstruct Pulmon Dis. (2023) 18:493–506. doi: 10.2147/COPD.S402122, 37056681 PMC10086390

[4] Stolz D Mkorombindo T Schumann DM Agusti A Ash SY Bafadhel M . Towards the elimination of chronic obstructive pulmonary disease: a lancet commission. Lancet. (2022) 400:921–72. doi: 10.1016/S0140-6736(22)01273-9, 36075255 PMC11260396

[5] Al Wachami N Guennouni M Iderdar Y Boumendil K Arraji M Mourajid Y . Estimating the global prevalence of chronic obstructive pulmonary disease (COPD): a systematic review and meta-analysis. BMC Public Health. (2024) 24:38273271:297. doi: 10.1186/s12889-024-17686-9 PMC10811845 38273271

[6] World Health Organization. World Health Statistics 2024: Monitoring Health for the SDGs, Sustainable Development Goals. Geneva: World Health Organization; (2024). Available online at: https://www.who.int/publications/i/item/9789240094703 (Accessed February 10, 2026).

[7] Wang Y Han R Ding X Feng W Gao R Ma A. Chronic obstructive pulmonary disease across three decades: trends, inequalities, and projections from the global burden of disease study 2021. Front Med. (2025) 12:1564878. doi: 10.3389/fmed.2025.1564878, 40196348 PMC11973060

[8] Wang Y Jin L Dong Y Yang E Niu X Mao J . Global burden of disease study on COPD in the older adult: comprehensive analysis of environmental factors and interaction effects. Front Public Health. (2025) 13:1597793. doi: 10.3389/fpubh.2025.1597793, 40520278 PMC12162481

[9] Troosters T Janssens W Demeyer H Rabinovich RA. Pulmonary rehabilitation and physical interventions. Eur Respir Rev. (2023) 32:220222. doi: 10.1183/16000617.0222-2022, 37286219 PMC10245142

[10] Zhang H Hu D Xu Y Wu L Lou L. Effect of pulmonary rehabilitation in patients with chronic obstructive pulmonary disease: a systematic review and meta-analysis of randomized controlled trials. Ann Med. (2022) 54:262–73. doi: 10.1080/07853890.2021.1999494, 35037535 PMC8765243

[11] Lamberton CE Mosher CL. Review of the evidence for pulmonary rehabilitation in COPD: clinical benefits and cost-effectiveness. Respir Care. (2024) 69:686–96. doi: 10.4187/respcare.11541, 38503466 PMC11147635

[12] Rochester CL Alison JA Carlin B Jenkins AR Cox NS Bauldoff G . Pulmonary rehabilitation for adults with chronic respiratory disease: an official American Thoracic Society clinical practice guideline. Am J Respir Crit Care Med. (2023) 208:e7–e26. doi: 10.1164/rccm.202306-1066ST, 37581410 PMC10449064

[13] Chen YH Chen LR Tsao CC Chen YC Huang CC. Effects of a pedometer-based walking program in patients with COPD-a pilot study. Medicina. (2022) 58:490. doi: 10.3390/medicina58040490, 35454330 PMC9026463

[14] Duruturk N Arıkan H Ulubay G Tekindal MA. A comparison of calisthenic and cycle exercise training in chronic obstructive pulmonary disease patients: a randomized controlled trial. Expert Rev Respir Med. (2016) 10:99–108. doi: 10.1586/17476348.2015.1126419, 26616764

[15] Xu W Li R Guan L Wang K Hu Y Xu L . Combination of inspiratory and expiratory muscle training in same respiratory cycle versus different cycles in COPD patients: a randomized trial. Respir Res. (2018) 19:225. doi: 10.1186/s12931-018-0917-6, 30458805 PMC6245535

[16] Wang L Wu K Chen X Wang LH Wu KL Chen XD . The effects of tai chi on lung function, exercise capacity and health related quality of life for patients with chronic obstructive pulmonary disease: a pilot study. Heart Lung Circ. (2019) 28:1206–12. doi: 10.1016/j.hlc.2018.05.204, 30166260

[17] Wu W Liu X Liu J Li P Wang Z. Effectiveness of water-based Liuzijue exercise on respiratory muscle strength and peripheral skeletal muscle function in patients with COPD. Int J Chron Obstruct Pulmon Dis. (2018) 13:1713–26. doi: 10.2147/COPD.S165593, 29872289 PMC5973471

[18] Shang X Yan X Ma Y. Comparing the effectiveness of single exercises on improving exercise capacity in chronic obstructive pulmonary disease patients: network meta-analysis of randomized controlled trials. Heart Lung. (2025) 70:278–92. doi: 10.1016/j.hrtlng.2025.01.003, 39798187

[19] Hutton B Salanti G Caldwell DM Chaimani A Schmid CH Cameron C . The PRISMA extension statement for reporting of systematic reviews incorporating network meta-analyses of health care interventions: checklist and explanations. Ann Intern Med. (2015) 162:777–84. doi: 10.7326/M14-2385, 26030634

[20] Global Initiative for Chronic Obstructive Lung Disease. Global strategy for the diagnosis, management, and prevention of chronic obstructive pulmonary disease: 2024 report. (2024). Available online at: https://goldcopd.org/2024-gold-report/ (Accessed February 10, 2026).

[21] Weir CJ Butcher I Assi V Lewis SC Murray GD Langhorne P . Dealing with missing standard deviation and mean values in meta-analysis of continuous outcomes: a systematic review. BMC Med Res Methodol. (2018) 18:25. doi: 10.1186/s12874-018-0483-0, 29514597 PMC5842611

[22] Higgins JP Altman DG Gøtzsche PC Jüni P Moher D Oxman AD . The Cochrane collaboration’s tool for assessing risk of bias in randomised trials. BMJ. (2011) 343:d5928. doi: 10.1136/bmj.d5928 22008217 PMC3196245

[23] Guyatt GH Oxman AD Kunz R Brozek J Alonso-Coello P Rind D . GRADE guidelines 6. Rating the quality of evidence--imprecision. J Clin Epidemiol. (2011) 64:1283–93. doi: 10.1016/j.jclinepi.2011.01.012, 21839614

[24] Melsen WG Bootsma MC Rovers MM Bonten MJ. The effects of clinical and statistical heterogeneity on the predictive values of results from meta-analyses. Clin Microbiol Infect. (2014) 20:123–9. doi: 10.1111/1469-0691.12494, 24320992

[25] Agusti A Ambrosino N Blackstock F Bourbeau J Casaburi R Celli B . COPD: providing the right treatment for the right patient at the right time. Respir Med. (2023) 207:107041. doi: 10.1016/j.rmed.2022.107041, 36610384

[26] Ries AL Bauldoff GS Carlin BW Casaburi R Emery CF Mahler DA . Pulmonary rehabilitation: joint ACCP/AACVPR evidence-based clinical practice guidelines. Chest. (2007) 131:4S–42S. doi: 10.1378/chest.06-2418, 17494825

[27] Backman H Vanfleteren LEGW Mannino DM Ekström M. Severity of airflow obstruction based on FEV1/FVC versus FEV1 percent predicted in the general U.S. population. Am J Respir Crit Care Med. (2024) 210:1308–16. doi: 10.1164/rccm.202310-1773OC, 38597717 PMC11622431

[28] Xiao L Duan H Li P Wu W Shan C Liu X. A systematic review and meta-analysis of Liuzijue in stable patients with chronic obstructive pulmonary disease. BMC Complement Med Ther. (2020) 20:308. doi: 10.1186/s12906-020-03104-1, 33054800 PMC7557061

[29] Liu P Li Y Tang D Liu G Zou Y Ma Y . Effects of different traditional Chinese exercises on pulmonary function in patients with stable chronic obstructive pulmonary disease: a network meta-analysis. BMC Complement Med Ther. (2024) 24:304. doi: 10.1186/s12906-024-04609-9, 39143580 PMC11325568

[30] Marinho-Buzelli AR Zaluski AJ Mansfield A Bonnyman AM Musselman KE. The use of aquatic therapy among rehabilitation professionals for individuals with spinal cord injury or disorder. J Spinal Cord Med. (2019) 42:158–65. doi: 10.1080/10790268.2019.1647935, 31573458 PMC6783731

[31] Becker BE. Aquatic therapy: scientific foundations and clinical rehabilitation applications. PM R. (2009) 1:859–72. doi: 10.1016/j.pmrj.2009.05.017, 19769921

[32] Khaltaev N Solimene U Vitale F Zanasi A. Balneotherapy and hydrotherapy in chronic respiratory disease. J Thorac Dis. (2020) 12:4459–68. doi: 10.21037/jtd-gard-2019-009, 32944359 PMC7475532

[33] Sánchez Romero EA Zubercová AJ Gran A de Abreu RM de Araujo FX Sinatti P . Comparative effectiveness of water-based versus land-based rehabilitation in COPD: a systematic review and network meta-analysis of randomized controlled trials. NPJ Prim Care Respir Med. (2026). doi: 10.1038/s41533-026-00503-8 PMC13427819 41965361

[34] Lareau S ZuWallack R Nici L. Increasing quality and quantity of life in individuals with chronic obstructive pulmonary disease: a narrative review with an emphasis on pulmonary rehabilitation. Life (Basel). (2025) 15:750. doi: 10.3390/life15050750, 40430178 PMC12112767

[35] Guo G Wang Y Xu X Lu K Zhu X Gu Y . Effectiveness of Yijinjing exercise in the treatment of early-stage knee osteoarthritis: a randomized controlled trial protocol. BMJ Open. (2024) 14:e074508. doi: 10.1136/bmjopen-2023-074508, 38453194 PMC10921529

[36] Zhang M Xu GH Li F Luo CF Meng SJ Zhi Y. Health Qigong Yijinjing promotes rehabilitation in patients with stable chronic obstructive pulmonary disease. Chin J Sports Med. (2016) 35:339–343.

[37] Zhang M Xv G Luo C Meng DJ Ji Y. Qigong Yi Jinjing promotes pulmonary function, physical activity, quality of life and emotion regulation self-efficacy in patients with chronic obstructive pulmonary disease: a pilot study. J Altern Complement Med. (2016) 22:810–7. doi: 10.1089/acm.2015.0224, 27487437

[38] Glenny AM Altman DG Song F Sakarovitch C Deeks J D'Amico R . Indirect comparisons of competing interventions. Health Technol Assess. (2005) 9:1–iv. doi: 10.3310/hta9260, 16014203

