# Supplementary material for: Comparative efficacy of single exercise interventions on pulmonary function and quality of life in patients with chronic obstructive pulmonary disease: a systematic review and network meta-analysis
Source: Front Med (Lausanne). 2026 May 28;13:1843777. doi: 10.3389/fmed.2026.1843777 (PMC13254076; doi:10.3389/fmed.2026.1843777)
Supplement: Supplementary file 1 [file Data_Sheet_1.docx]

**Supplementary Material**

**Appendix 1 Detailed searching strategies**

**1.1 Search strategy in PubMed（n = 2195）**

| #1 | (((((((((("Pulmonary Disease, Chronic Obstructive"[Mesh]) OR (Chronic Obstructive Pulmonary Diseases[Title/Abstract])) OR (COPD[Title/Abstract])) OR (Chronic Obstructive Lung Disease[Title/Abstract])) OR (Chronic Obstructive Pulmonary Disease[Title/Abstract])) OR (COAD[Title/Abstract])) OR (Chronic Obstructive Airway Disease[Title/Abstract])) OR (Airflow Obstruction, Chronic[Title/Abstract])) OR (Airflow Obstructions, Chronic[Title/Abstract])) OR (Chronic Airflow Obstructions[Title/Abstract])) OR (Chronic Airflow Obstruction[Title/Abstract]) |
| --- | --- |
| #2 | ((((((((((((((((((((((((("Exercise"[Mesh]) OR (Exercises[Title/Abstract])) OR (Exercise, Physical[Title/Abstract])) OR (Exercises, Physical[Title/Abstract])) OR (Physical Exercise[Title/Abstract])) OR (Physical Exercises[Title/Abstract])) OR (Exercise, Aerobic[Title/Abstract])) OR (Aerobic Exercise[Title/Abstract])) OR (Aerobic Exercises[Title/Abstract])) OR (Exercises, Aerobic[Title/Abstract])) OR (Exercise, Isometric[Title/Abstract])) OR (Exercises, Isometric[Title/Abstract])) OR (Isometric Exercises[Title/Abstract])) OR (Isometric Exercise[Title/Abstract])) OR (Acute Exercise[Title/Abstract])) OR (Acute Exercises[Title/Abstract])) OR (Exercise, Acute[Title/Abstract])) OR (Exercises, Acute[Title/Abstract])) OR (Exercise Training[Title/Abstract])) OR (Exercise Trainings[Title/Abstract])) OR (Training, Exercise[Title/Abstract])) OR (Trainings, Exercise[Title/Abstract])) OR (Physical Activity[Title/Abstract])) OR (Activities, Physical[Title/Abstract])) OR (Activity, Physical[Title/Abstract])) OR (Physical Activities[Title/Abstract]) |
| #3 | ((((((((((((((((Walking[Title/Abstract]) OR (walk training[Title/Abstract])) OR (Cycling[Title/Abstract])) OR (Cycle exercise[Title/Abstract])) OR (breathing training[Title/Abstract])) OR (Respiratory muscle training[Title/Abstract])) OR (Inspiratory muscle training[Title/Abstract])) OR (Expiratory muscle training[Title/Abstract])) OR (Diaphragmatic training[Title/Abstract])) OR (Qigong[Title/Abstract])) OR (Yoga[Title/Abstract])) OR (Tai chi[Title/Abstract])) OR (Taiji[Title/Abstract])) OR (Baduanjin[Title/Abstract])) OR (Yijinjing[Title/Abstract])) OR (Wuqinxi[Title/Abstract])) OR (Liuzijue[Title/Abstract]) |
| #4 | ((((((("Randomized Controlled Trials as Topic"[Mesh]) OR (Clinical Trials, Randomized)) OR (Trials, Randomized Clinical)) OR (Controlled Clinical Trials, Randomized)) OR (RCT)) OR (random[Title/Abstract])) OR (randomized[Title/Abstract])) OR (randomised[Title/Abstract]) |
| #5 | #2 OR #3 |
| #6 | #1 AND #4 AND #5 |

**1.2 Search strategy in Embase（n = 305）**

| #1 | 'pulmonary disease, chronic obstructive':ti,ab,kw OR 'chronic obstructive pulmonary diseases':ti,ab,kw OR copd:ti,ab,kw OR 'chronic obstructive lung disease':ti,ab,kw OR 'chronic obstructive pulmonary disease':ti,ab,kw OR coad:ti,ab,kw OR 'chronic obstructive airway disease':ti,ab,kw OR 'airflow obstruction, chronic':ti,ab,kw OR 'airflow obstructions, chronic':ti,ab,kw OR 'chronic airflow obstructions':ti,ab,kw OR 'chronic airflow obstruction':ti,ab,kw |
| --- | --- |
| #2 | exercise:ti,ab,kw OR exercises:ti,ab,kw OR 'exercise, physical':ti,ab,kw OR 'exercises, physical':ti,ab,kw OR 'physical exercise':ti,ab,kw OR 'physical exercises':ti,ab,kw OR 'exercise, aerobic':ti,ab,kw OR 'aerobic exercise':ti,ab,kw OR 'aerobic exercises':ti,ab,kw OR 'exercises, aerobic':ti,ab,kw OR 'exercise, isometric':ti,ab,kw OR 'exercises, isometric':ti,ab,kw OR 'isometric exercises':ti,ab,kw OR 'isometric exercise':ti,ab,kw OR 'acute exercise':ti,ab,kw OR 'acute exercises':ti,ab,kw OR 'exercise, acute':ti,ab,kw OR 'exercises, acute':ti,ab,kw OR 'exercise training':ti,ab,kw OR 'exercise trainings':ti,ab,kw OR 'training, exercise':ti,ab,kw OR 'trainings, exercise':ti,ab,kw OR 'physical activity':ti,ab,kw OR 'activities, physical':ti,ab,kw OR 'activity, physical':ti,ab,kw OR 'physical activities':ti,ab,kw |
| #3 | walking:ti,ab,kw OR 'walk training':ti,ab,kw OR cycling:ti,ab,kw OR 'cycle exercise':ti,ab,kw OR 'breathing training':ti,ab,kw OR 'respiratory muscle training':ti,ab,kw OR 'inspiratory muscle training':ti,ab,kw OR 'expiratory muscle training':ti,ab,kw OR 'diaphragmatic training':ti,ab,kw OR qigong:ti,ab,kw OR yoga:ti,ab,kw OR 'tai chi':ti,ab,kw OR taiji:ti,ab,kw OR baduanjin:ti,ab,kw OR yijinjing:ti,ab,kw OR wuqinxi:ti,ab,kw OR liuzijue:ti,ab,kw |
| #4 | 'randomized controlled trials as topic':ti,ab,kw OR 'clinical trials, randomized':ti,ab,kw OR 'trials, randomized clinical':ti,ab,kw OR 'controlled clinical trials, randomized':ti,ab,kw OR rct:ti,ab,kw |
| #5 | #2 OR #3 |
| #6 | #1 AND #4 AND #5 |

**1.3 Search strategy in Web of science（n = 1019）**

| #1 | ((((((((((TS=(Pulmonary Disease, Chronic Obstructive)) OR TS=(Chronic Obstructive Pulmonary Diseases)) OR TS=(COPD)) OR TS=(Chronic Obstructive Lung Disease)) OR TS=(Chronic Obstructive Pulmonary Disease)) OR TS=(COAD)) OR TS=(Chronic Obstructive Airway Disease)) OR TS=(Airflow Obstruction, Chronic)) OR TS=(Airflow Obstructions, Chronic)) OR TS=(Chronic Airflow Obstructions)) OR TS=(Chronic Airflow Obstruction) |
| --- | --- |
| #2 | (((((((((((((((((((((((((TS=(Exercise)) OR TS=(Exercises)) OR TS=(Exercise, Physical)) OR TS=(Exercises, Physical)) OR TS=(Physical Exercise)) OR TS=(Physical Exercises)) OR TS=(Exercise, Aerobic)) OR TS=(Aerobic Exercise)) OR TS=(Aerobic Exercises)) OR TS=(Exercises, Aerobic)) OR TS=(Exercise, Isometric)) OR TS=(Exercises, Isometric)) OR TS=(Isometric Exercises)) OR TS=(Isometric Exercise)) OR TS=(Acute Exercise)) OR TS=(Acute Exercises)) OR TS=(Exercise, Acute)) OR TS=(Exercises, Acute)) OR TS=(Exercise Training)) OR TS=(Exercise Trainings)) OR TS=(Training, Exercise)) OR TS=(Trainings, Exercise)) OR TS=(Physical Activity)) OR TS=(Activities, Physical)) OR TS=(Activity, Physical)) OR TS=(Physical Activities) |
| #3 | ((((((((((((((((TS=(Walking)) OR TS=(walk training)) OR TS=(Cycling)) OR TS=(Cycle exercise)) OR TS=(breathing training)) OR TS=(Respiratory muscle training)) OR TS=(Inspiratory muscle training)) OR TS=(Expiratory muscle training)) OR TS=(Diaphragmatic training)) OR TS=(Qigong)) OR TS=(Yoga)) OR TS=(Tai chi)) OR TS=(Taiji)) OR TS=(Baduanjin)) OR TS=(Yijinjing)) OR TS=(Wuqinxi)) OR TS=(Liuzijue) |
| #4 | ((((TS=(Randomized Controlled Trials as Topic)) OR TS=(Clinical Trials, Randomized)) OR TS=(Trials, Randomized Clinical)) OR TS=(Controlled Clinical Trials, Randomized)) OR TS=(RCT) |
| #5 | #2 OR #3 |
| #6 | #1 AND #4 AND #5 |

**1.4 Search strategy in Cochrane Library（n = 707）**

| #1 | (Pulmonary Disease, Chronic Obstructive):ti,ab,kw OR (Chronic Obstructive Pulmonary Diseases):ti,ab,kw OR (COPD):ti,ab,kw OR (Chronic Obstructive Lung Disease):ti,ab,kw OR (Chronic Obstructive Pulmonary Disease):ti,ab,kw |
| --- | --- |
| #2 | (COAD):ti,ab,kw OR (Chronic Obstructive Airway Disease):ti,ab,kw OR (Airflow Obstruction, Chronic):ti,ab,kw OR (Airflow Obstructions, Chronic):ti,ab,kw OR (Chronic Airflow Obstructions):ti,ab,kw |
| #3 | (Chronic Airflow Obstruction):ti,ab,kw |
| #4 | #1 OR #2 OR #3 |
| #5 | (Exercise):ti,ab,kw OR (Exercises):ti,ab,kw OR (Exercise, Physical):ti,ab,kw OR (Exercises, Physical):ti,ab,kw OR (Physical Exercise):ti,ab,kw |
| #6 | (Physical Exercises):ti,ab,kw OR (Exercise, Aerobic):ti,ab,kw OR (Aerobic Exercise):ti,ab,kw OR (Aerobic Exercises):ti,ab,kw OR (Exercises, Aerobic):ti,ab,kw |
| #7 | (Exercise, Isometric):ti,ab,kw OR (Exercises, Isometric):ti,ab,kw OR (Isometric Exercises):ti,ab,kw OR (Isometric Exercise):ti,ab,kw OR (Acute Exercise):ti,ab,kw |
| #8 | (Acute Exercises):ti,ab,kw OR (Exercise, Acute):ti,ab,kw OR (Exercises, Acute):ti,ab,kw OR (Exercise Training):ti,ab,kw OR (Exercise Trainings):ti,ab,kw |
| #9 | (Training, Exercise):ti,ab,kw OR (Trainings, Exercise):ti,ab,kw OR (Physical Activity):ti,ab,kw OR (Activities, Physical):ti,ab,kw OR (Activity, Physical):ti,ab,kw |
| #10 | (Physical Activities):ti,ab,kw |
| #11 | #5 OR #6 OR #7 OR #8 OR #9 OR #10 |
| #12 | (Walking):ti,ab,kw OR (Walk training):ti,ab,kw OR (Cycling):ti,ab,kw OR (Cycle exercise):ti,ab,kw OR (Breathing training):ti,ab,kw |
| #13 | (Respiratory muscle training):ti,ab,kw OR (Inspiratory muscle training):ti,ab,kw OR (Expiratory muscle training):ti,ab,kw OR (Diaphragmatic training):ti,ab,kw OR (Qigong):ti,ab,kw |
| #14 | (Yoga):ti,ab,kw OR (Tai chi):ti,ab,kw OR (Taiji):ti,ab,kw OR (Baduanjin):ti,ab,kw OR (Yijinjing):ti,ab,kw |
| #15 | (Wuqinxi):ti,ab,kw OR (Liuzijue):ti,ab,kw |
| #16 | #12 OR #13 OR #14 OR #15 |
| #17 | #11 OR #16 |
| #18 | (Randomized Controlled Trials as Topic):ti,ab,kw OR (Clinical Trials, Randomized):ti,ab,kw OR (Trials, Randomized Clinical):ti,ab,kw OR (Controlled Clinical Trials, Randomized):ti,ab,kw OR (RCT):ti,ab,kw |
| #19 | #4 AND #17 AND #18 |

**1.5 Search strategy in Scopus（n = 555）**

| #1 | TITLE-ABS-KEY ("Pulmonary Disease, Chronic Obstructive" OR "Chronic Obstructive Pulmonary Diseases" OR "COPD" OR "Chronic Obstructive Lung Disease" OR "Chronic Obstructive Pulmonary Disease" OR "COAD" OR "Chronic Obstructive Airway Disease" OR "Airflow Obstruction, Chronic" OR "Airflow Obstructions, Chronic" OR "Chronic Airflow Obstructions" OR "Chronic Airflow Obstruction") |
| --- | --- |
| #2 | TITLE-ABS-KEY ("exercise" OR "exercises" OR "exercise, physical" OR "exercises, physical" OR "physical exercise" OR "physical exercises" OR "exercise, aerobic" OR "aerobic exercise" OR "aerobic exercises" OR "exercises, aerobic" OR "exercise, isometric" OR "exercises, isometric" OR "isometric exercises" OR "isometric exercise" OR "acute exercise" OR "acute exercises" OR "exercise, acute" OR "exercises, acute" OR "exercise training" OR "exercise trainings" OR "training, exercise" OR "trainings, exercise" OR "physical activity" OR "activities, physical" OR "activity, physical" OR "physical activities") |
| #3 | TITLE-ABS-KEY ("Walking" OR "Walk training" OR "Cycling" OR "Cycle exercise" OR "breathing training" OR "Respiratory muscle training" OR "Inspiratory muscle training" OR "Expiratory muscle training" OR "Diaphragmatic training" OR "Qigong" OR "Yoga" OR "Tai chi" OR "Taiji" OR "Baduanjin" OR "Yijinjing" OR "Wuqinxi" OR "Liuzijue") |
| #4 | TITLE-ABS-KEY ("Randomized Controlled Trials as Topic" OR "Clinical Trials, Randomized" OR "Trials, Randomized Clinical" OR "Controlled Clinical Trials, Randomized" OR "RCT") |
| #5 | #2 OR #3 |
| #6 | #1 AND #4 AND #5 |

**1.6 Search strategy in CNKI（n = 4644）**

| #1 | 主题=（慢性阻塞性肺疾病 + 慢阻肺 + COPD） AND 主题=（运动 + 步行 + 散步 + 自行车 + 呼吸肌训练 + 吸气肌训练 + 呼气肌训练 + 膈肌训练 + 气功 + 瑜伽 + 太极拳 + 八段锦 + 易筋经 + 五禽戏 + 六字诀） |
| --- | --- |

**Appendix 2 Data processing**

If a study reports only baseline and change means and SD, the endpoint mean is calculated as the sum of the baseline mean and change mean.

| Baseline | Change | Endpoint |
| --- | --- | --- |
| Mean (B)  SD (B) | Mean (C)  SD (C) | Mean (E)  SD (E) |

Mean (E)= Mean (B) + Mean (C)


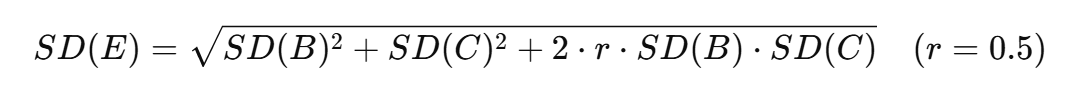


For studies reporting medians and interquartile ranges (IQRs), the median was approximated as the mean and the SD was estimated as IQR/1.35.

For studies reporting means and standard errors (SE) instead of standard deviations (SD), we converted SE to SD using the formula


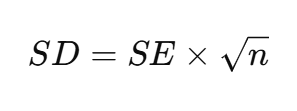


**Appendix 3 The basic characteristics of included studies**

| Study | Country | Sample size  (EG/CG) | Age  (years, EG/CG) | FEV_1_%pred  (%, EG/CG) | Interventions  (EG/CG) | frequency and duration | outcomes |
| --- | --- | --- | --- | --- | --- | --- | --- |
| Yu 2003[1] | China | 72(36/36) | NA | 42.5±18.4/43.0±16.4 | Walking/CT | 30 min/time, 2-3 times/day, 2 months | ① |
| Li 2015[2] | China | 100(50/50) | 60.5 ± 4.8 | NA | Walking/CT | ≥45min/time, 3 months | ② |
| Duan 2016[3] | China | 86(46/40) | 66.7 ± 6.6/67.4 ± 7.1 | NA | Walking/CT | 6 min/time, once/day, 12 months | ② |
| Jin 2016[4] | China | 78(40/38) | 67.3 ± 6.8/68.8 ± 7.1 | 42.26±7.32/42.65±7.84 | Walking/CT | 6 min/time, 2 times/day, 3 months | ①② |
| Chen 2022[5] | China | 26(15/11) | 73.5 ± 8.2/71.9 ± 11.1 | 51.2±13.7/53.0±16.3 | Walking/CT | 7 days/week, 6 weeks | ① |
| Cui 2011[6] | China | 35(20/15) | 65.9 ± 5.8/67.6 ± 6.5 | 54.9 ± 14.4/51.4 ± 12.1 | Cycling/CT | 30 min/time, 3 times/week, 16 weeks | ① |
| Zhang 2012[7] | China | 29(19/10) | 67.2 ± 4.5/67.1 ± 4.3 | 47.0±12.7/43.8±14.0 | Cycling/CT | 40 min/time, 2 times/week, 8 weeks | ① |
| Wu 2015[8] | China | 30(15/15) | 61 ± 7/60 ± 8 | 39±8/37±9 | Cycling/CT | 30 min/time, 3 times/week, 12 weeks | ①② |
| Duruturk 2016[9] | Turkey | 31(15/13) | 61.2 ± 5.0/63.8 ± 5.7 | 58.4±14.4/63.6±10.8 | Cycling/CT | 20-45 min/time, 3 times/week, 6 weeks | ① |
| Wang(1) 2018[10] | China | 192(89/103) | 52.25 ± 3.18/52.83 ± 2.32 | 48.1±11.3/48.8±12.7 | Cycling/CT | 20–30 min/time, 3 months, 3–5 times/week | ① |
| Scherer 2000[11] | Switzerland | 30(15/15) | 66.9 ± 2.4/71.0 ± 1.2 | 50.2±17.04/52.3±13.56 | RMT/CT | 15 min/time, 2 times/day, 5 days/week, 8 weeks | ① |
| Hill 2006[12] | Australia | 33(16/17) | 69.4 ± 7.2/66.6 ± 9.8 | 37.4±12.5/36.5±11.5 | RMT/CT | 21 min/time, 3 times/week, 8 weeks | ① |
| Bavarsad 2015[13] | Iran | 30(15/15) | 58.8 ± 6.82/54.2 ± 8.09 | 62.53±27.95/63.33±22.72 | RMT/CT | 15 min/day, 6 days/week, 8 weeks | ① |
| Wu 2017[14] | China | 39(19/20) | 59.74 ± 6.14/60.30 ± 6.55 | 54.2±6.27/54.5±7.13 | RMT/CT | 15 min/time, 2 times/day, 8 weeks | ① |
| Leelarungrayub 2017[15] | Thailand | 20(10/10) | 63.25 ± 1.49/68.75 ± 2.39 | 59.38±4.65/61.04±6.93 | RMT/CT | 20–30 min/time, once/day, 6 weeks | ① |
| Xu 2018[16] | China | 46(23/23) | 67.49 ± 6.17/69.43 ± 6.44 | 46.65±13.51/48.22±15.03 | RMT/CT | 48 min/day, 7 days/week, 8 weeks | ①② |
| Saka 2021[17] | Turkey | 40(20/20) | 62.30 ± 7.43/62.10 ± 7.76 | 39.30±12.37/40.50±14.92 | RMT/CT | 15 min/time, 2 times/day, 5 days/week, 8 weeks | ①② |
| Pulsakowska 2016[18] | Poland | 30(8/9/13) | 63.4 ± 9.8/62.3 ± 5.2/65.5 ± 7.0 | 49.9±17.2/49.0±17.1/68.4±20.0 | RMT/Cycling/CT | 20 min/time, 2 times/day, 6 weeks | ① |
| Du 2013[19] | China | 74(36/38) | 65.24 ± 8.37/64.48 ± 6.54 | 73.36±6.33/72.97±6.46 | Taichi/CT | 30 min/time, 2 times/day, 12 weeks | ①② |
| Zhang 2014[20] | China | 36(18/18) | 68.02 ± 6.91/66.71 ± 5.84 | 56.71±7.62/57.81±6.94 | Taichi/CT | 60 min/time, 2 times/day, 12 months | ①② |
| Li 2016[21] | China | 40(20/20) | 60.3 ± 6.9 | 46±16.5/46.1±16.5 | Taichi/CT | 40 min/time, 3 times/week, 3 months | ① |
| Pan 2018[22] | China | 41(20/21) | NA | 52.80±3.12/52.05±3.27 | Taichi/CT | 30 min/time, 3 times/week, 8 weeks | ①② |
| Zhu 2018[23] | China | 60(30/30) | 67.87 ± 5.22/68.10 ± 6.57 | 35.11±13.74/40.77±15.60 | Taichi/CT | 40-50 min/time, 3 times/week, 3 months | ①② |
| Liu 2019[24] | China | 100(50/50) | 53.5 ± 6.1/53.1 ± 5.6 | 47.81±2.30/49.60±3.82 | Taichi/CT | 30 min/time, 2 times/day, 12 months | ①② |
| Wang 2019[25] | China | 50(26/24) | 67.83 ± 5.32/67.86 ± 5.98 | 55.46±11.47/62.55±20.18 | Taichi/CT | 60 min/time, 3 times/week, 3 months | ①② |
| Peng 2020[26] | China | 80(40/40) | NA | NA | Taichi/CT | 30 min/time, once/day, 24 weeks | ② |
| Ni 2017[27] | China | 90(30/30/30) | 63.3 ± 2.2/65.3 ± 2.1/60.6 ± 4.5 | 57.12±9.7/58.12±10.50/8.91±9.60 | Taichi/Yoga/CT | 30 min/day, 6 months | ① |
| Chen 2008[28] | China | 40(21/19) | 71.76 ± 7.31/73.32 ± 6.33 | 41.13±15.74/41.37±16.77 | LBL/CT | 3 months | ① |
| Lan 2016[29] | China | 84(42/42) | 67.24 ± 3.21/67.02 ± 3.48 | 55.05±6.49/54.60±6.71 | LBL/CT | 60 min/time, 2 times/day, 5 days/week, 12 weeks | ①② |
| Shen 2017[30] | China | 100(50/50) | 72.15 ± 2.08/72.55 ± 2.15 | 56.83±3.51/56.99±3.12 | LBL/CT | 20 min/time, once/day, 6 months | ①② |
| Li 2018[31] | China | 36(17/19) | 66 ± 9/66 ± 9 | 55.50±16.8/58.49±19.4 | LBL/CT | 60 min/time, 6 times/week, 6 months | ① |
| Wu(1) 2018[32] | China | 33(16/17) | 67 ± 8/66 ± 9 | 55±17/55±16 | LBL/CT | 40 min/time, 6 times/week, 6 months | ① |
| Wu(2) 2018[33] | China | 31(15/16) | 65 ± 8/66 ± 8 | 55±17/59±17 | LBL/CT | 60 min/time, 2 times/week, 3 months | ① |
| Ji 2019[34] | China | 57(28/29) | 63.75 ± 5.48/64.52 ± 5.68 | 40.32±12.64/46.85±11.80 | LBL/CT | 30 min/time, 5 times/day, 3 months | ① |
| Shi 2020[35] | China | 60(30/30) | 64.34 ± 5.32/65.24 ± 5.22 | NA | LBL/CT | 30 min/time, once/day, 3 months | ② |
| Deng(1) 2020[36] | China | 62(30/32) | 76.53 ± 8.59/76.59 ± 7.69 | 57.66±26.17/54.24±23.24 | LBL/CT | 30 min/time, once/day, 3 months | ①② |
| Lu 2021[37] | China | 274(135/139) | NA | NA | LBL/CT | 20 min/time, once/day, 12 weeks | ② |
| Hu 2021[38] | China | 60(20/20/20) | NA | 50.09±11.61/49.36±10.42/50.02±12.48 | LBL/WBL/CT | 30 min/time, 3 times/day, 6 months | ①② |
| Yan 2021[39] | China | 40(20/20) | 64.89 ± 7.54/66.05 ± 8.65 | 56.34±15.20/58.46±15.21 | LBL/CT | 6 times/week, 6 months | ① |
| Fang 2022[40] | China | 60(30/30) | 68.72 ± 5.43/68.23 ± 5.27 | 52.64±5.12/52.78±5.23 | LBL/CT | 40 min/time, 5 times/week, 3 months | ① |
| Chen(1) 2024[41] | China | 64(32/32) | 63.68 ± 12.86/63.89 ± 13.02 | NA | LBL/CT | 30 min/time, 2 times/day, 3 months | ② |
| Xu 2025[42] | China | 140(70/70) | 67.0 ± 8.8/66.4 ± 8.6 | 40.90±27.07/47.9±20.52 | LBL/CT | 30 min/time, once/day, 24 weeks | ①② |
| Cao 2022[43] | China | 62(31/31) | 69.78 ± 0.68/69.30 ± 2.83 | 49.28±10.39/50.11±12.38 | WBL/CT | 30 min, 8 times/month, 6 months | ① |
| Zhu 2010[44] | China | 47(26/21) | 53.53 ± 10.05/55.12 ± 11.41/55.46 ± 9.87 | 55.02±10.87/53.07±12.02 | Wuqinxi/Walking/CT | 45 min/time, once/day, 3 months | ① |
| Wei 2015[45] | China | 93(48/45) | 58.66 ± 7.56/58.64 ± 7.52 | 64.91±8.72/64.55±6.03 | Wuqinxi/CT | 30 min/time, 1-2 times/day, ≥4 times/week, 6 months | ① |
| Cheng 2015[46] | China | 93(48/45) | 58.66 ± 7.56/58.64 ± 7.52 | NA | Wuqinxi/CT | 30 min/time, 1-2 times/day, ≥4 times/week, 6 months | ② |
| Zhao 2015[47] | China | 60(30/30) | 58.91 ± 5.86/56.66 ± 6.43 | 55.68±4.69/57.46±4.31 | Wuqinxi/CT | 40 min/time, 3 times/day, 3 months | ① |
| Gao 2017[48] | China | 72(36/36) | 67.14 ± 9.08/66.03 ± 8.18 | 43.03±8.94/44.51±10.58 | Wuqinxi/CT | 30 min/time, 2 times/day, 3 months | ① |
| Liu 2020[49] | China | 100(50/50) | 74.24 ± 9.10/67.72 ± 9.26 | 44.76±15.33/51.58±17.58 | Wuqinxi/CT | 45 min/time, once/day, 3 months | ① |
| Feng 2009[50] | China | 60(30/30) | 62 ± 5/63 ± 4 | 56.98±6.41/55.85±6.43 | Baduanjin/CT | 45 min/time, 2 times/day, 24 weeks | ① |
| Liu 2013[51] | China | 80(40/40) | 59.77 ± 7.08/60.67 ± 6.95 | 52.16±16.18/53.39±20.19 | Baduanjin/CT | 30 min/time, once/day, 3 months | ① |
| Zhu 2014[52] | China | 123(63/60) | 69.0 ± 8.7/68.0 ± 9.2 | 48.20±11.28/48.45±10.34 | Baduanjin/CT | 30 min/time, 2 times/day, 6 months | ① |
| Chen 2015[53] | China | 61(31/30） | 66.26 ± 5.13/66.90 ± 4.63 | NA | Baduanjin/CT | 30 min/time, once/day, 3 months | ② |
| Deng 2015[54] | China | 64(32/32) | 66.26 ± 5.13/66.90 ± 4.63 | 42.18±17.99/43.36±17.56 | Baduanjin/CT | 30 min/time, once/day, 3 months | ① |
| Liang 2016[55] | China | 82(41/41) | NA | 42.87±17.40/43.02±17.37 | Baduanjin/CT | 30 min/time, once/day, 3 months | ① |
| Pan 2016[56] | China | 84(42/42) | 60.7 ± 5.6/61.8 ± 7.2 | 53.78±13.73/53.03±12.94 | Baduanjin/CT | 30 min/time, once/day, 6 months | ① |
| Guo(1) 2016[57] | China | 60(30/30) | 62.8/63.4 | 54.84±5.39/56.49 ± 4.16 | Baduanjin/CT | 15-20 min/time, once/day, ≥4 times/week, 6 months | ① |
| Guo(2) 2016[58] | China | 320(161/159) | 64.15 ± 8.97/64.87 ± 8.86 | 54.11±19.97/53.87±19.89 | Baduanjin/CT | 30 min/time, once/day, ≥4 times/week, 6 months | ①② |
| Huang 2017[59] | China | 62(31/31) | 68.24 ± 3.28/69.77 ± 4.42 | 49.23±9.21/50.28±8.67 | Baduanjin/CT | 30 min/time, once/day, 6 months | ①② |
| Zhang 2017[60] | China | 60(30/30) | 68.50 ± 9.18/68.03 ± 7.92 | 52.40±9.96/52.55±7.76 | Baduanjin/CT | 2 months | ① |
| Zhu 2017[61] | China | 215(106/109) | 67.00 ± 8.70/68.00 ± 9.20 | NA | Baduanjin/CT | 40 min/time, 2 times/day, 6 months | ① |
| Wang(2) 2018[62] | China | 76(38/38) | 63.17 ± 9.95/63.67 ± 9.75 | 53.36±8.31/53.27±8.02 | Baduanjin/CT | 30 min/time, once/day, 3 months | ① |
| Dong 2018[63] | China | 92(46/46) | 63.97 ± 5.57/64.25 ± 6.01 | 50.23±8.96/49.85±8.74 | Baduanjin/CT | 30 min/time, once/day, ≥4 times/week, 6 months | ①② |
| Zhang 2019[64] | China | 60(30/30) | 65.46 ± 6.74/64.82 ± 6.23 | 65.98±6.49/66.11±6.51 | Baduanjin/CT | 30 min/time, once/day, 6 months | ① |
| Yu 2019[65] | China | 82(41/41) | 62.3 ± 1.2/62.3 ± 1.5 | 52.42±3.84/52.34±3.27 | Baduanjin/CT | 30 min/time, once/day, 3 months | ① |
| Deng(2) 2020[66] | China | 54(27/27) | 64.84 ± 9.03/63.92 ± 8.47 | 50.15±20.38/49.85±16.54 | Baduanjin/CT | 30 min/time, once/day, ≥4 times/week, 6 months | ① |
| Liu 2021[67] | China | 70(35/35) | 59.85 ± 15.83/60.25 ± 16.35 | NA | Baduanjin/CT | 30 min/time, once/day, 4 weeks | ② |
| Feng 2024[68] | China | 84(42/42) | 53.01 ± 3.20/52.36 ± 3.14 | NA | Baduanjin/CT | 40 min/time, once/day, 6 weeks | ② |
| Chen(2) 2024[69] | China | 318(158/160） | 61.52 ± 10.31/61.97 ± 10.91 | NA | Baduanjin/CT | 30 min/time, 2 times/day, ≥5 days/week, 6 months | ② |
| Chen 2025[70] | China | 108(55/53） | 66.7 ± 6.7/66.7 ± 8.1 | 46.45±20.97/46.77±13.64 | Baduanjin/CT | 30 min/time, 2 times/day, 5 days/week, 12 weeks | ①② |
| Zhang 2025[71] | China | 96(48/48) | NA | 47.95±0.38/48.0±0.45 | Baduanjin/CT | 10–20 min/time, 2 times/day, 5 days/week, 2 months | ①② |
| Zhang(1) 2016[72] | China | 45(20/25) | 61.77 ± 4.07/59.35 ± 5.27 | 65.46±3.54/62.02±4.27 | Yijinjing/CT | 60 min/time, 2 times/day, 6 months | ① |
| Zhang(2) 2016[73] | China | 87(42/45) | 64.77 ± 11.07/62.35 ± 9.27 | 59.12±4.13/58.11±4.37 | Yijinjing/CT | 60 min/time, once/day, 6 months | ①② |
| Donesky 2009[74] | USA | 29(14/15) | 72.2 ± 6.5/67.7 ± 11.5 | 51.2±10.5/44.4±19.0 | Yoga/CT | 60 min/time, 2 times/week, 12 weeks | ① |
| Gupta 2014[75] | India | 50(25/25) | 52.5 ± 3.9/52 ± 4.1 | 51.1±8.7/49.6±8.6 | Yoga/CT | 30 min/time, 2 times/day, 3 months | ①② |
| Kaminsky 2017[76] | USA | 43(21/22) | 68 ± 7/68 ± 9 | 43±16/42±13 | Yoga/CT | 30 min/time, once/day, 12 weeks | ①② |
| Yudawhati 2019[77] | Indonesia | 30(15/15) | 64.40 ± 10.45/65.33 ± 8.12 | 43.53±20.63/40.87±22.31 | Yoga/CT | 30 min/time, 2 times/week, 12 weeks | ① |

EG = experimental group, CG = control group, CT = conventional treatment, NA = not available, RMT = respiratory Muscle Training, LBL = Land-based Liuzijue, WBL = Water-based Liuzijue, ①FEV_1_%, ②CAT. The relevant abbreviations in the following figures or tables have the same meaning.

**Appendix 4** Result of the risk of bias assessment

**
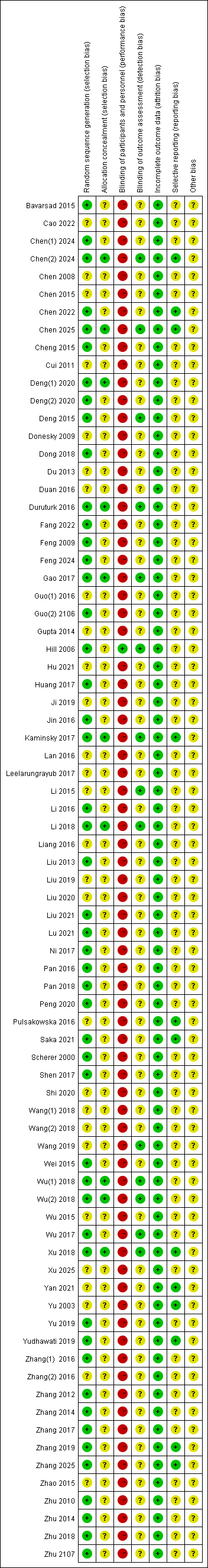
**

**Appendix 5 GRADE Assessment**

| Outcomes | Risk of Bias | Inconsistency | Indirectness | Imprecision | Other Considerations | Quality |
| --- | --- | --- | --- | --- | --- | --- |
| FEV_1_%pred | serious^1^ | no | no | serious^2^ | no | low |
| CAT | serious^1^ | no | no | serious^2^ | no | low |

^1^ There is a potential risk of bias in the inclusion of studies in terms of randomization, allocation concealment, and blinding methods.
^2^ The confidence intervals are wide and the total sample size is relatively small

**Appendix 6 Assessment of inconsistency**

**6.1 Global inconsistency test**

|  | Chi²(2) | *P* |
| --- | --- | --- |
| FEV_1_%pred | 8.00 | 0.433 |
| CAT | 1.34 | 0.997 |

**6.2 Node-splitting method**

**6.2.1 Node-splitting method for FEV_1_%pred**

| **Side** | **Direct** | | **Indirect** | | **Difference** | | | **tau** |
| --- | --- | --- | --- | --- | --- | --- | --- | --- |
|  | **Coefficient** | **SE** | **Coefficient** | **SE** | **Coefficient** | **SE** | **P> \| z \|** |  |
| Walking-Wuqinxi | 6.17 | 6.02 | 4.83 | 3.92 | 1.34 | 7.20 | 0.852 | 4.31 |
| Walking-CT | -3.36 | 2.70 | 5.51 | 10.38 | -8.86 | 10.68 | 0.406 | 4.25 |
| Cycling-RMT | 3.03 | 9.53 | -5.41 | 3.27 | 8.44 | 10.10 | 0.403 | 4.26 |
| Cycling-CT | -4.54 | 2.43 | -16.04 | 18.21 | 11.50 | 18.39 | 0.532 | 4.29 |
| RMT-CT | 0.10 | 2.03 | -28.37 | 15.41 | 28.48 | 15.47 | 0.066 | 4.20 |
| Taichi-Yoga | 0.92 | 5.31 | 3.51 | 3.67 | -2.59 | 6.42 | 0.687 | 4.32 |
| Taichi-CT | -1.91 | 1.81 | -4.05 | 11.32 | 2.14 | 11.47 | 0.852 | 4.32 |
| LBL-WBL | 3.71 | 4.54 | 2.23 | 4.90 | 1.48 | 6.60 | 0.822 | 4.32 |
| LBL-CT | -6.42 | 1.67 | -9.62 | 13.12 | 3.20 | 13.23 | 0.809 | 4.32 |
| WBL-CT | -8.93 | 3.43 | -13.17 | 8.42 | 4.24 | 8.98 | 0.637 | 4.31 |
| Wuqinxi-CT | -8.26 | 2.27 | -1.32 | 11.83 | -6.94 | 11.99 | 0.562 | 4.31 |
| Yoga-CT | -4.96 | 2.74 | -0.31 | 9.68 | -4.64 | 9.98 | 0.642 | 4.32 |

**6.2.2 Node-splitting method for CAT**

| **Side** | **Direct** | | **Indirect** | | **Difference** | | | **tau** |
| --- | --- | --- | --- | --- | --- | --- | --- | --- |
|  | **Coefficient** | **SE** | **Coefficient** | **SE** | **Coefficient** | **SE** | **P> \| z \|** |  |
| LBL-WBL | -1.61 | 2.12 | -1.59 | 4.16 | -0.02 | 4.70 | 0.997 | 1.93 |
| LBL-CT | 2.41 | 0.73 | 7.55 | 66.29 | -5.14 | 66.30 | 0.938 | 1.88 |
| WBL-CT | 4.01 | 2.18 | 4.03 | 4.06 | -0.02 | 4.70 | 0.996 | 1.93 |

**6.3 Loop inconsistency**

| Outcome | Loop | IF | SeIF | Z_value | P_value | CI_95 | Loop_Heterog_tau |
| --- | --- | --- | --- | --- | --- | --- | --- |
| FEV_1_%pred | Cycling-RMT-CT | 2.762 | 10.524 | 0.262 | 0.793 | (0.00, 23.39) | 23.110 |
| FEV_1_%pred | RMT-Yoga-CT | 2.372 | 6.594 | 0.360 | 0.719 | (0.00, 15.30) | 21.453 |
| FEV_1_%pred | Walking-Wuqinxi-CT | 1.676 | 11.088 | 0.151 | 0.880 | (0.00, 23.41) | 44.797 |
| FEV_1_%pred | LBL-WBL-CT | 1.008 | 5.277 | 0.191 | 0.849 | (0.00, 11.35) | 11.327 |
| CAT | LBL-WBL-CT | 0.081 | 2.273 | 0.036 | 0.971 | (0.00, 4.54) | 1.467 |

**Appendix 7 Pairwise meta-analysis forest plots**

**7.1 Pairwise meta-analysis forest plots on FEV_1_%pred**

**7.1.1 Pairwise meta-analysis forest plots of Walking on FEV_1_%pred**

**
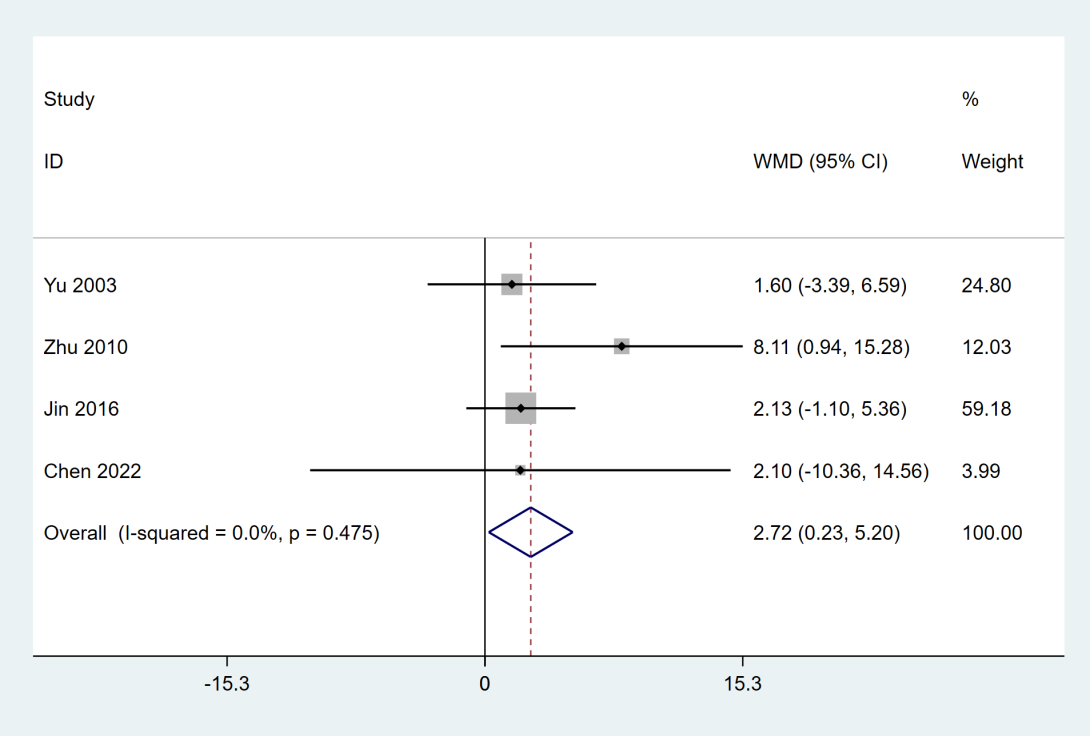
**

**7.1.2 Pairwise meta-analysis forest plots of Cycling on FEV_1_%pred**

**
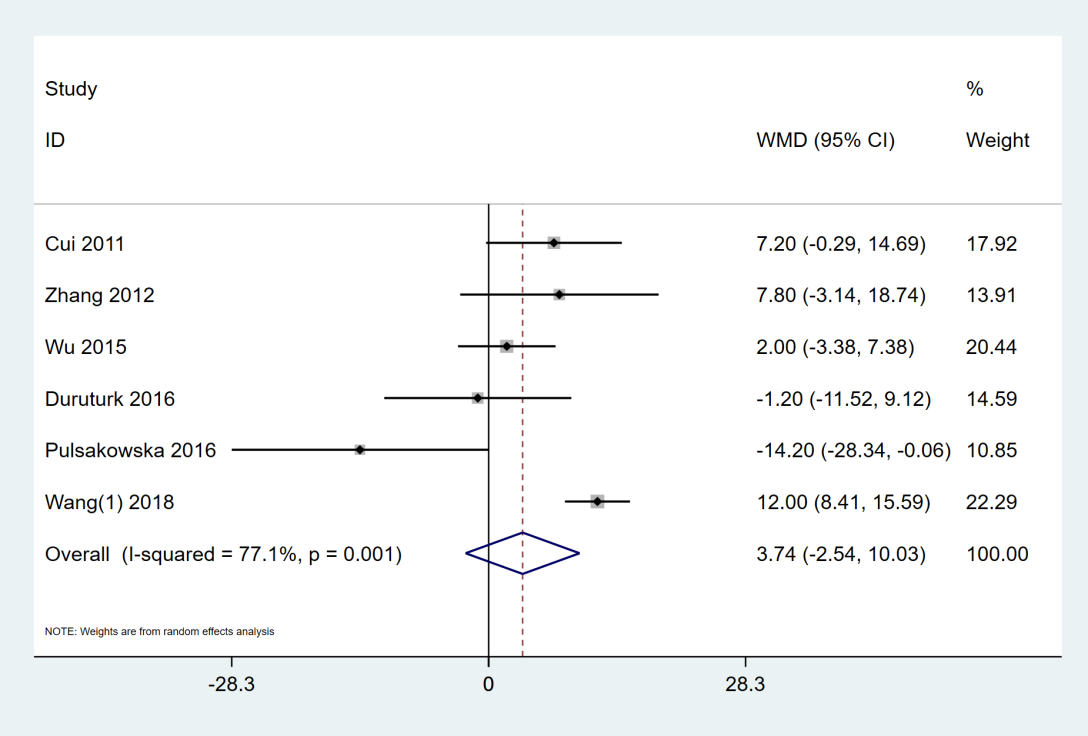
**

**7.1.3 Pairwise meta-analysis forest plots of RMT on FEV_1_%pred**

**
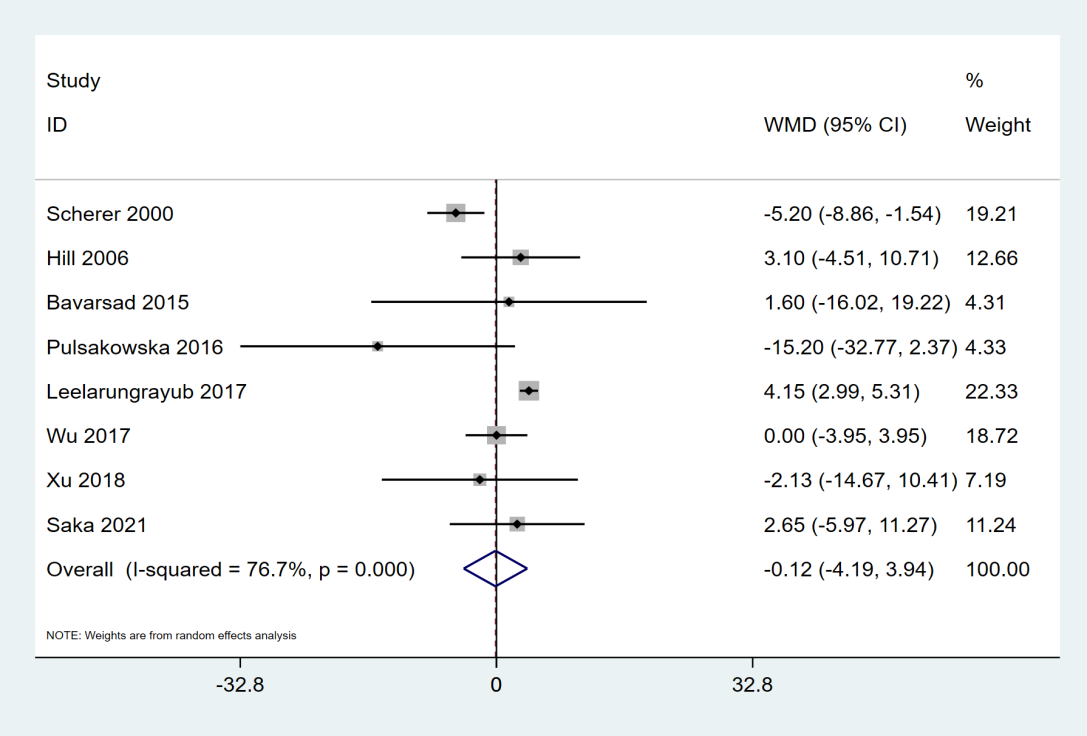
**

**7.1.4 Pairwise meta-analysis forest plots of Taichi on FEV_1_%pred**

**
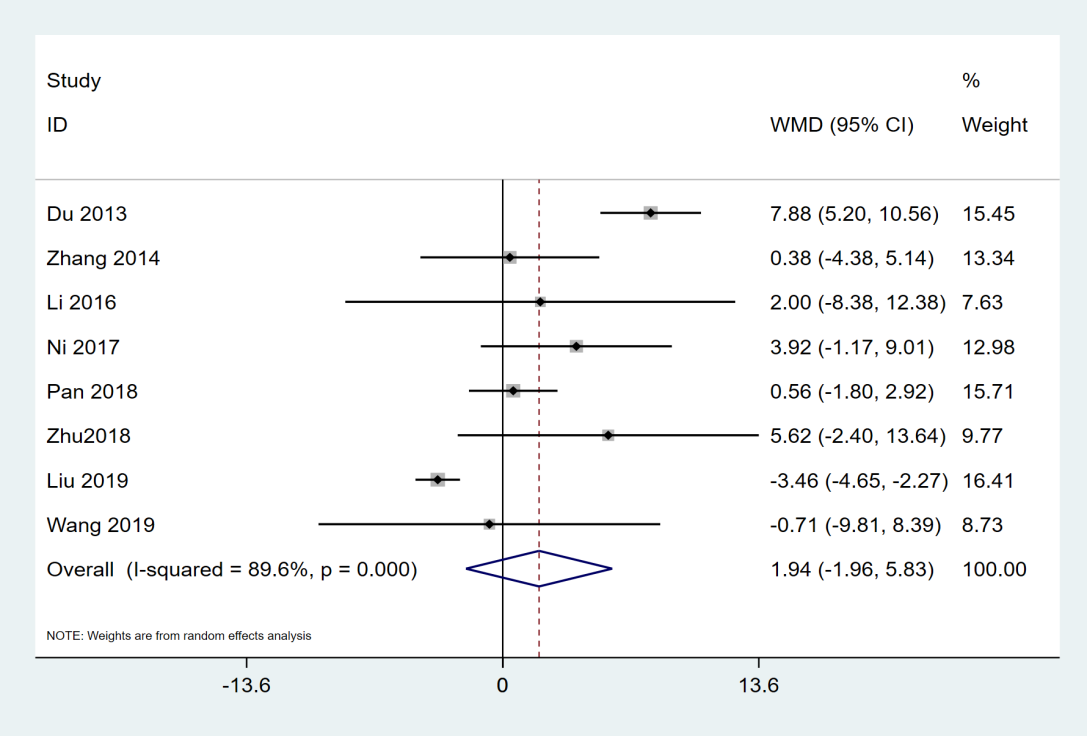
**

**7.1.5 Pairwise meta-analysis forest plots of LBL on FEV_1_%pred**

**
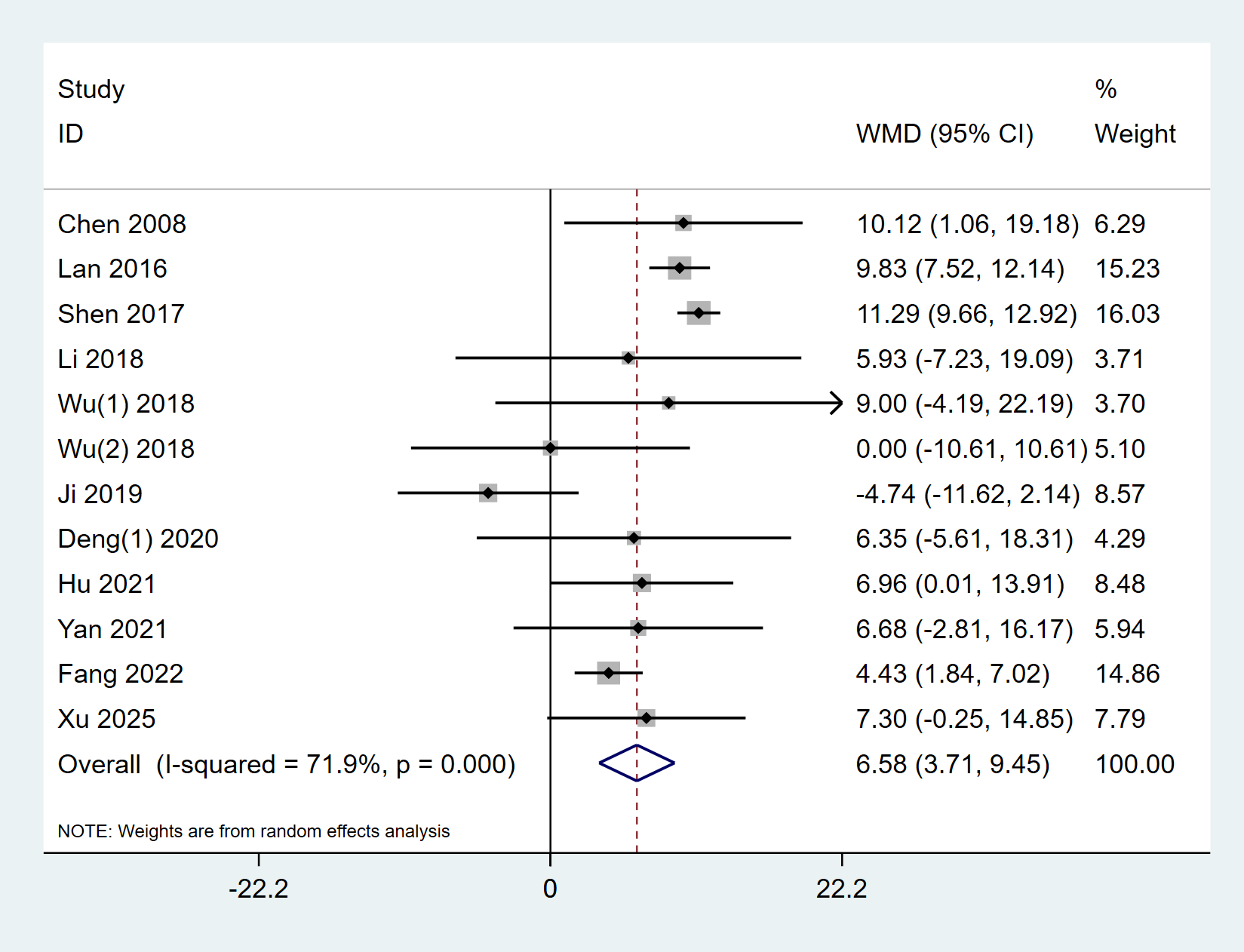
**

**7.1.6 Pairwise meta-analysis forest plots of WBL on FEV_1_%pred**

**
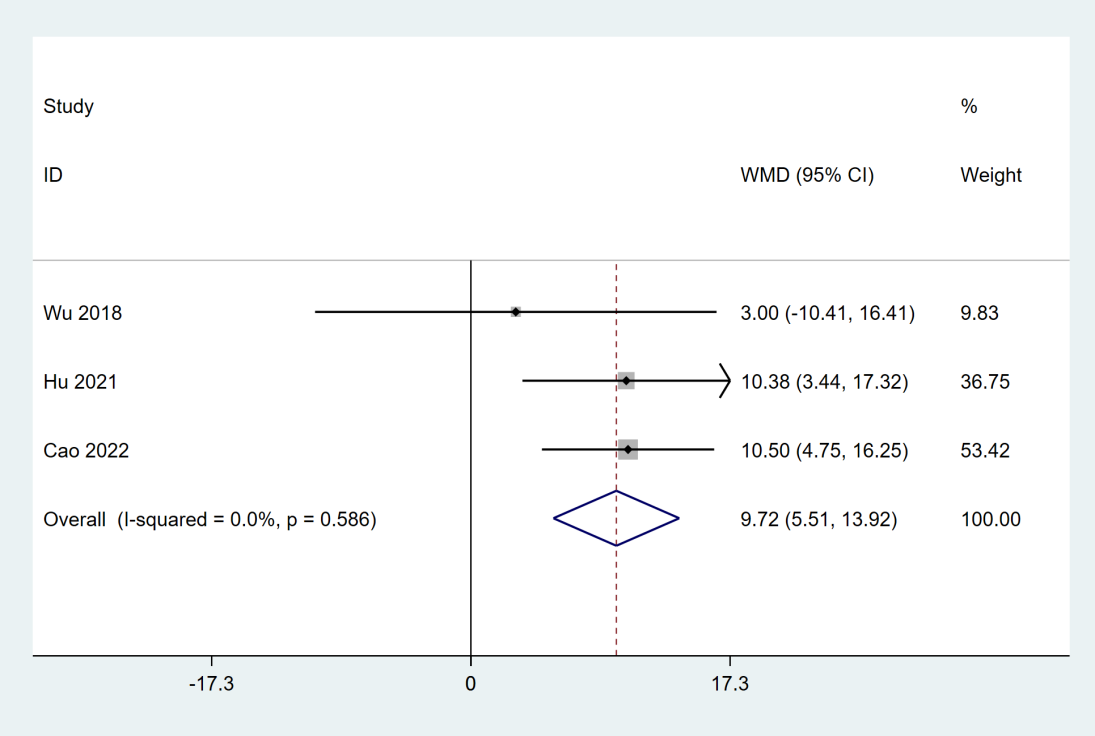
**

**7.1.7 Pairwise meta-analysis forest plots of Wuqinxi on FEV_1_%pred**

**
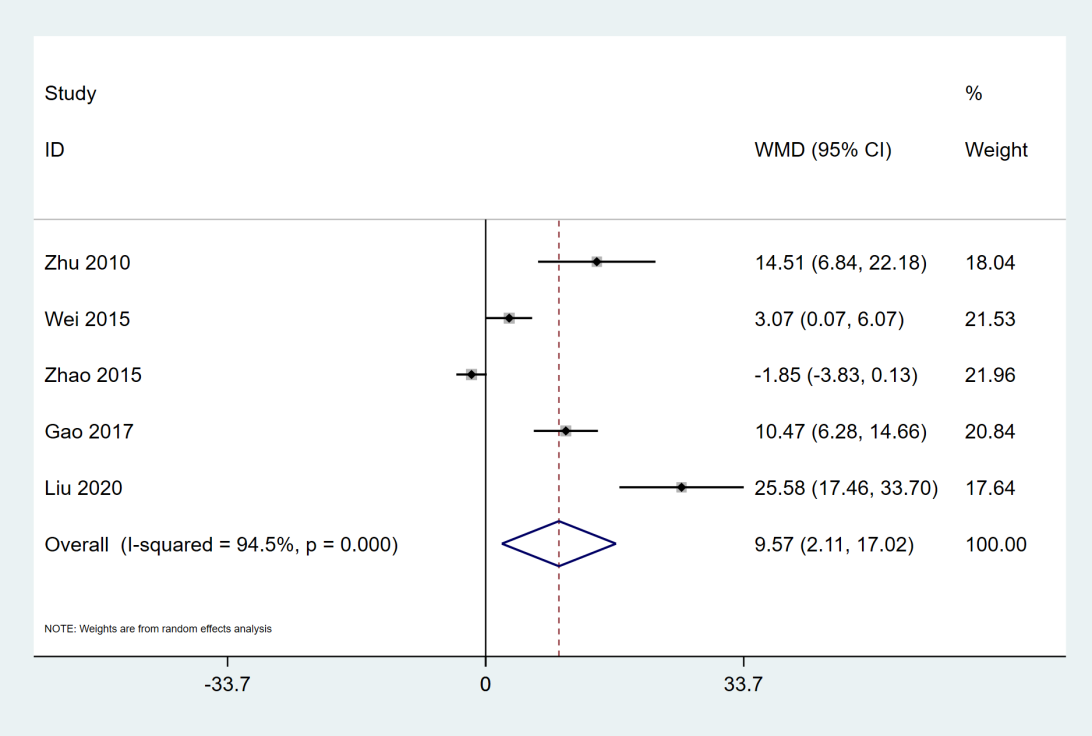
**

**7.1.8 Pairwise meta-analysis forest plots of Baduanjin on FEV_1_%pred**

**
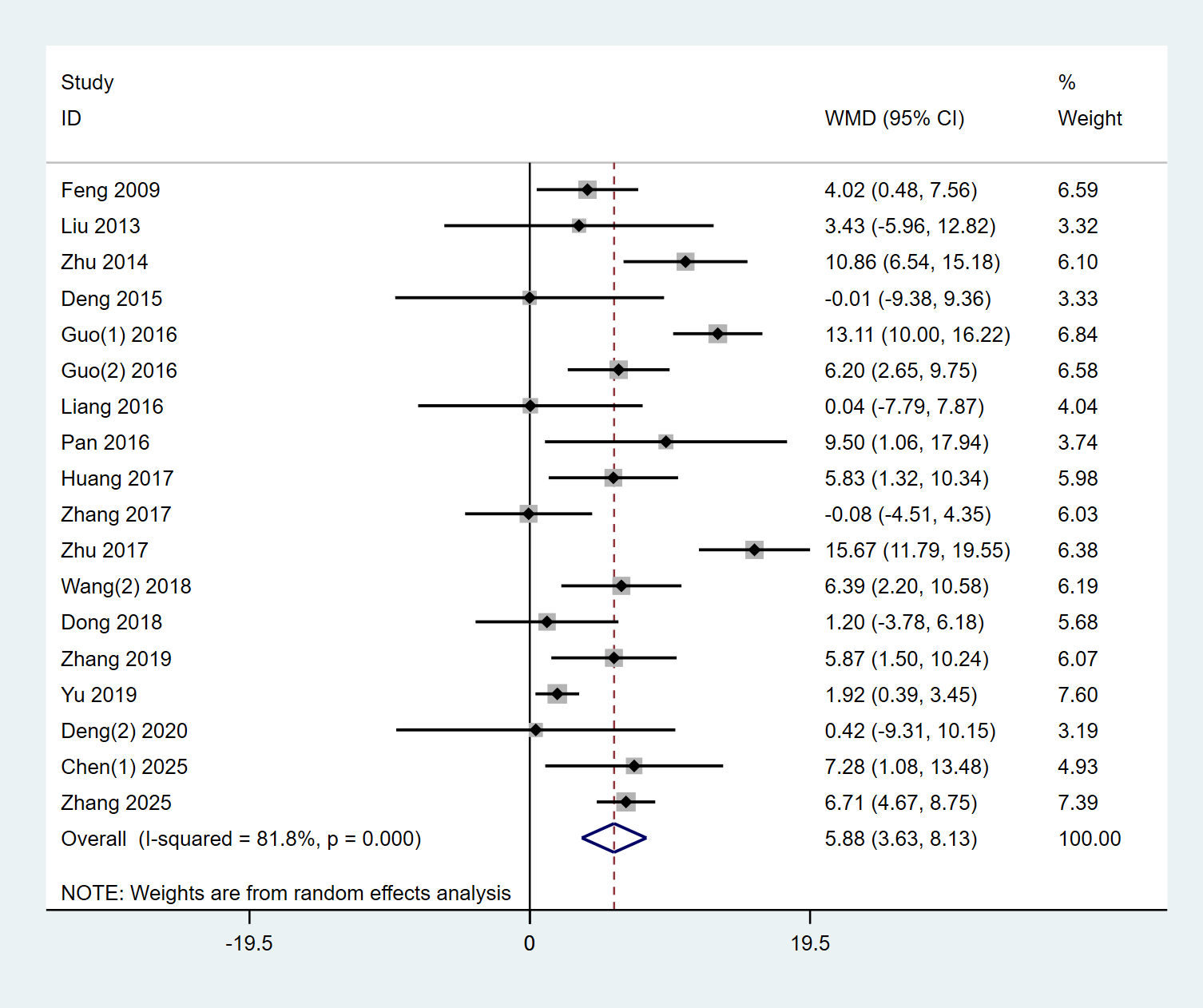
**

**7.1.9 Pairwise meta-analysis forest plots of Yijinjing on FEV_1_%pred**

**
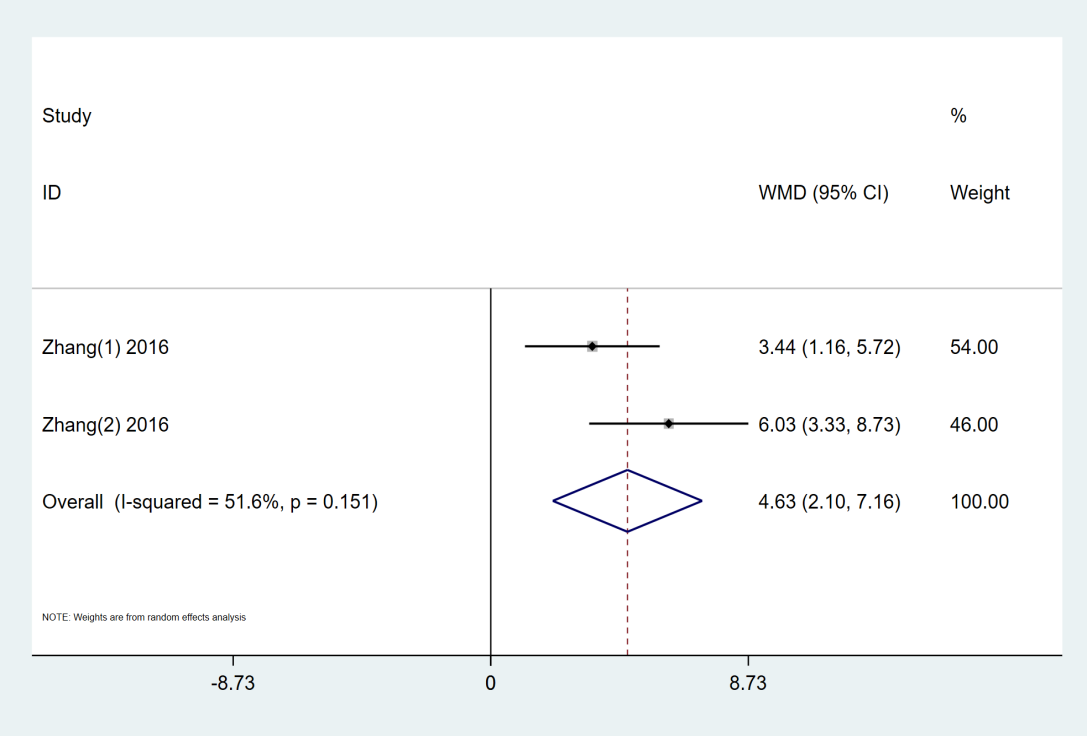
**

**7.1.10 Pairwise meta-analysis forest plots of Yoga on FEV_1_%pred**

**
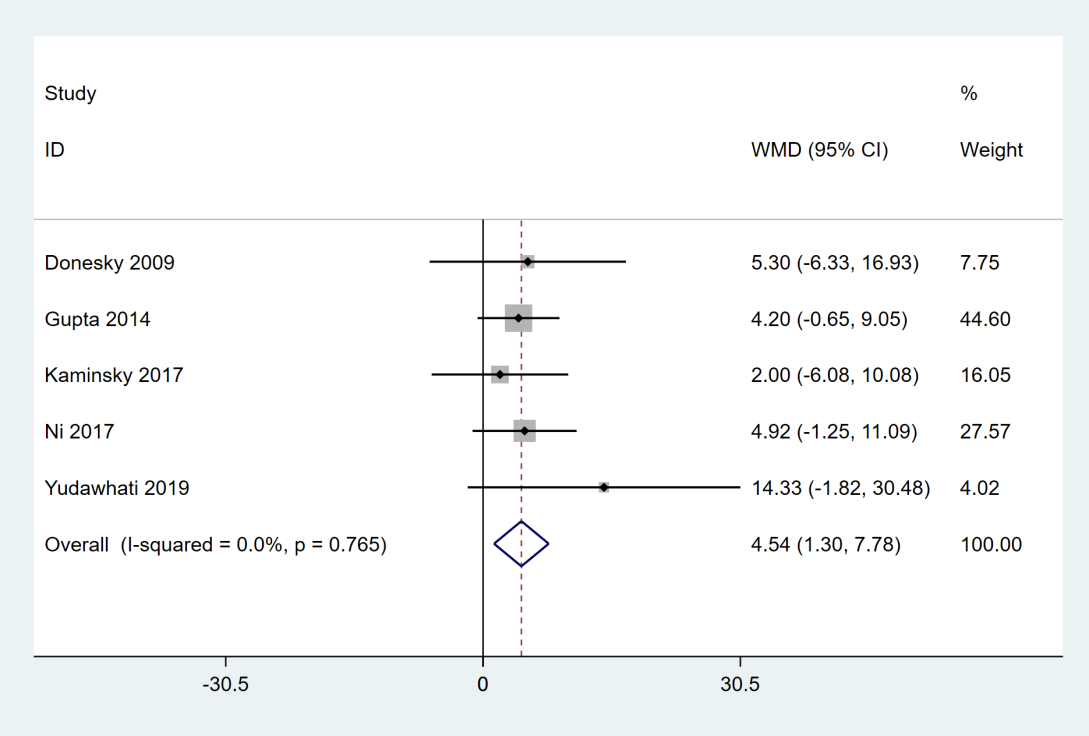
**

**7.2 Pairwise meta-analysis forest plots on CAT**

**7.2.1 Pairwise meta-analysis forest plots of Walking on CAT**

**
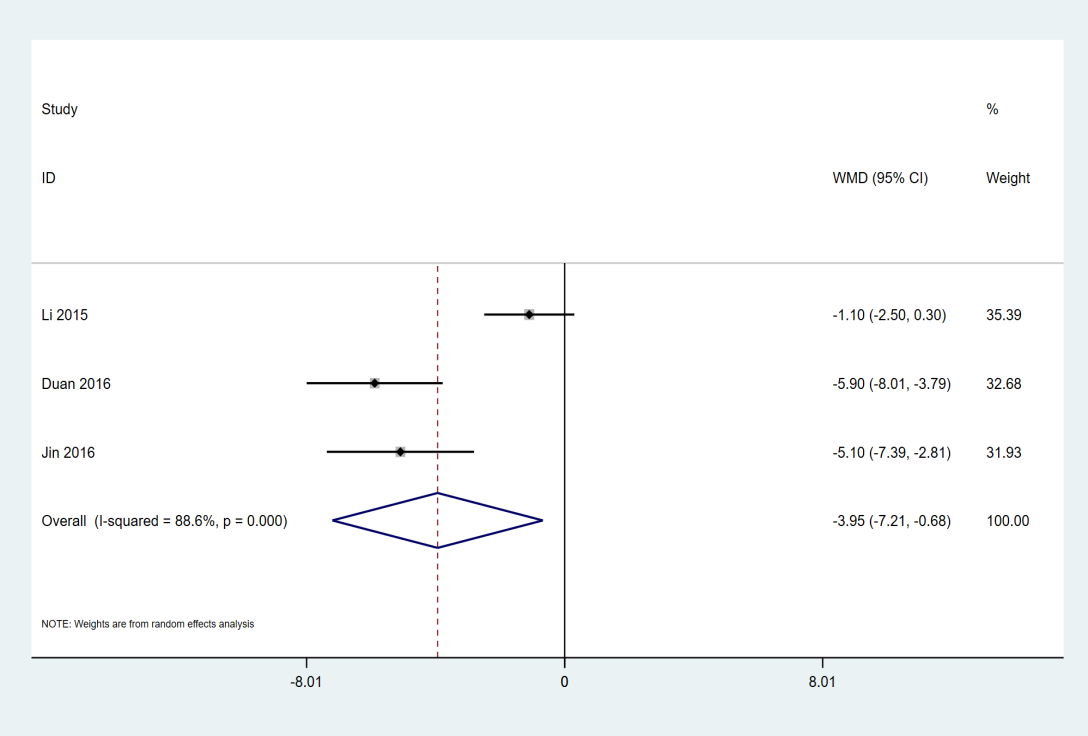
**

**7.2.2 Pairwise meta-analysis forest plots of RMT on CAT**

**
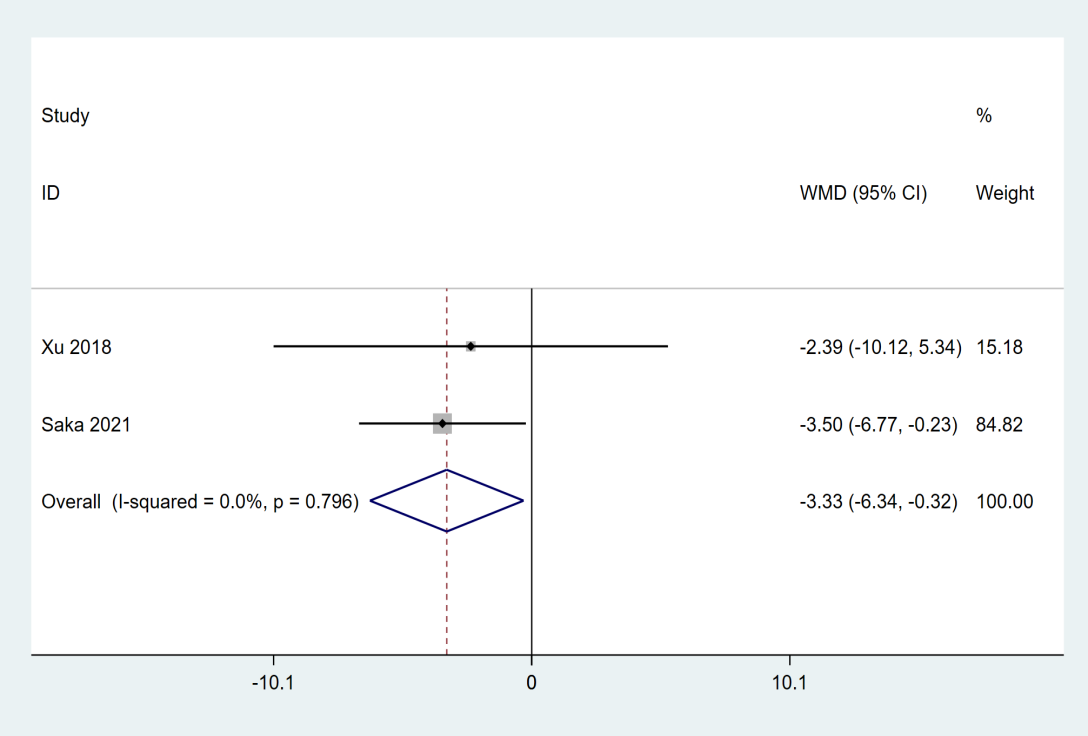
**

**7.2.3 Pairwise meta-analysis forest plots of Taichi on CAT**

**
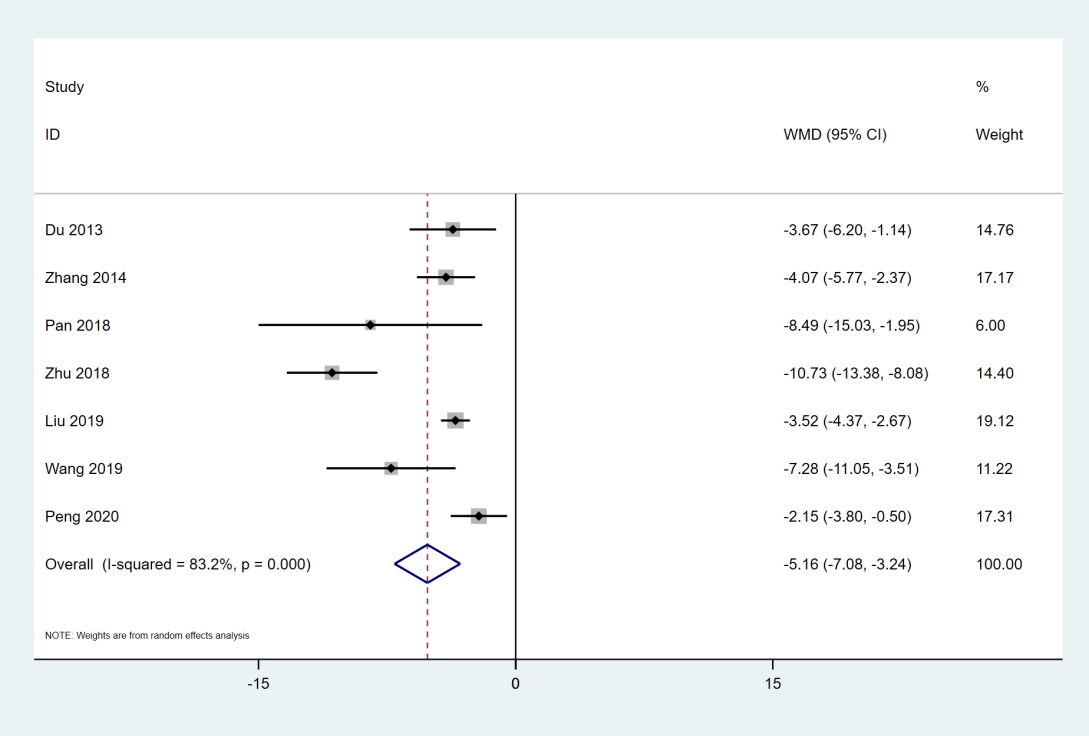
**

**7.2.4 Pairwise meta-analysis forest plots of LBL on CAT**

**
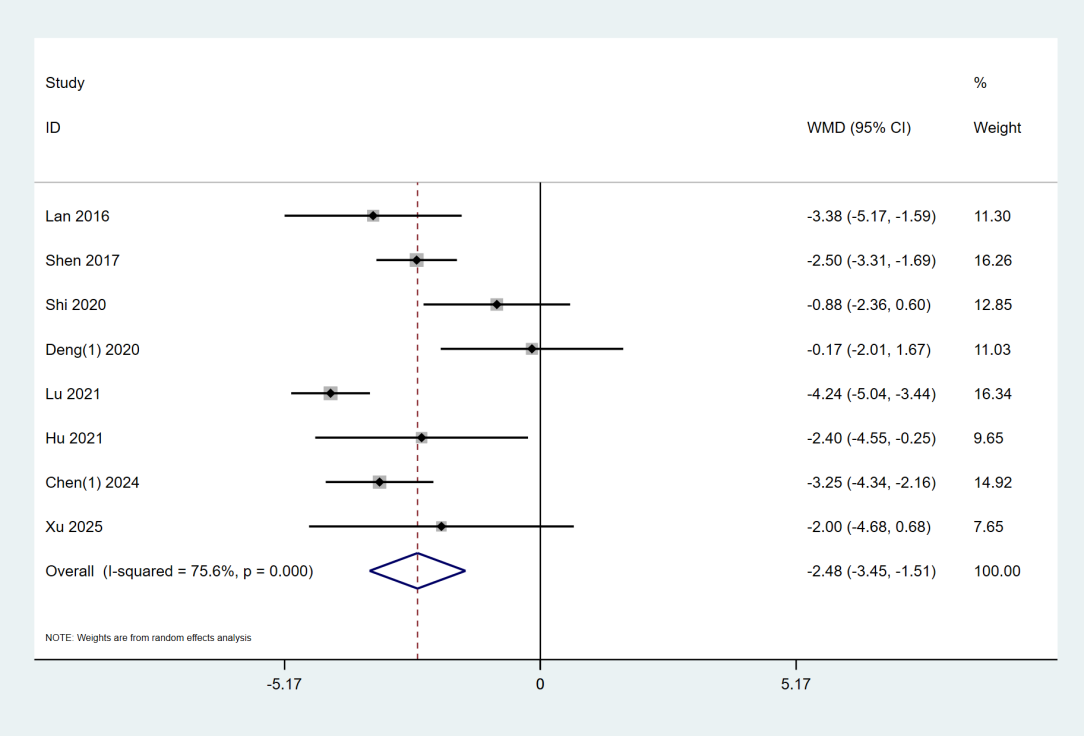
**

**7.2.5 Pairwise meta-analysis forest plots of Baduanjin on CAT**

**
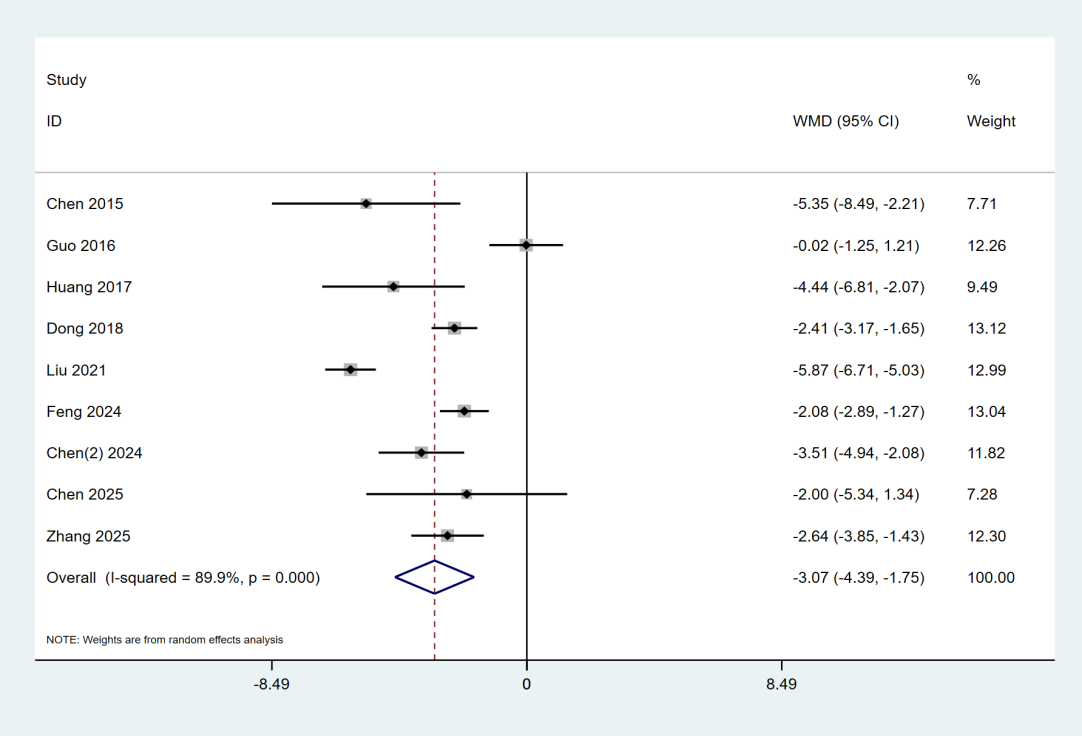
**

**7.2.6 Pairwise meta-analysis forest plots of Yoga on CAT**

**
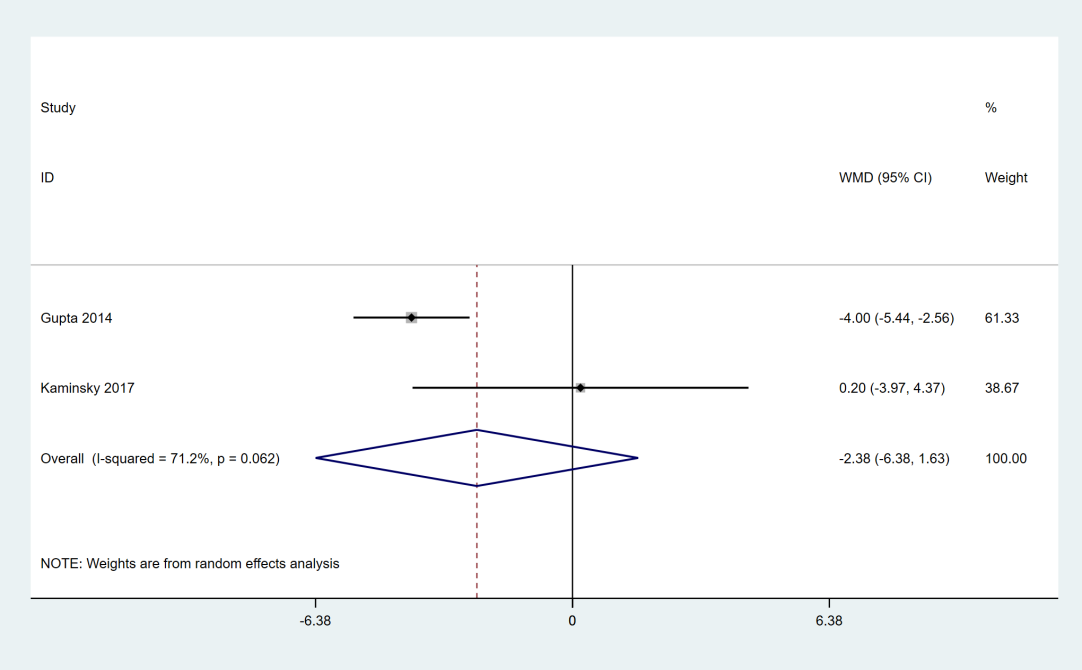
**

**Appendix 8 Network meta-analysis forest plots**

**8.1 Network meta-analysis forest plots on FEV_1_%pred**

**
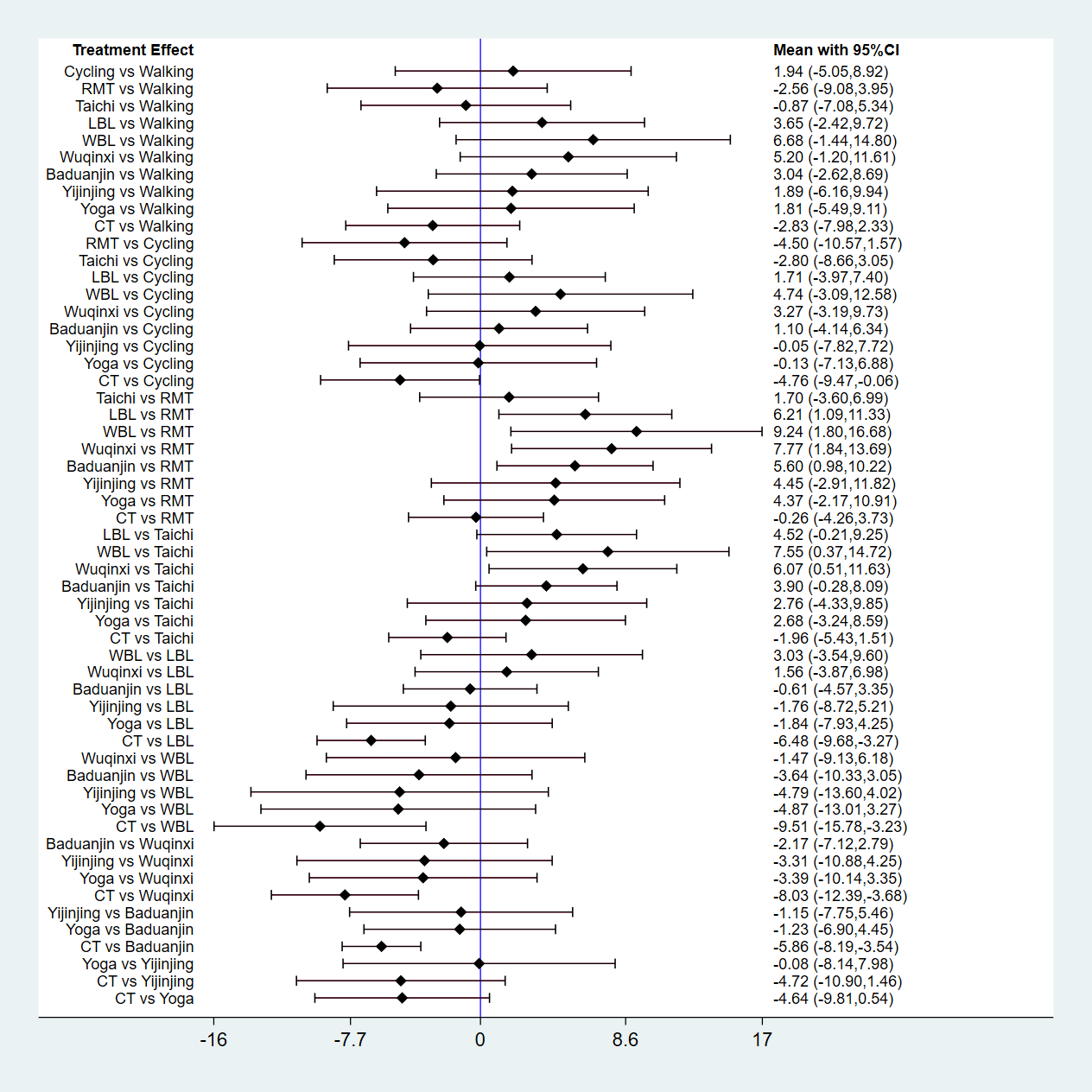
**

**8.2 Network meta-analysis forest plots on CAT**

**
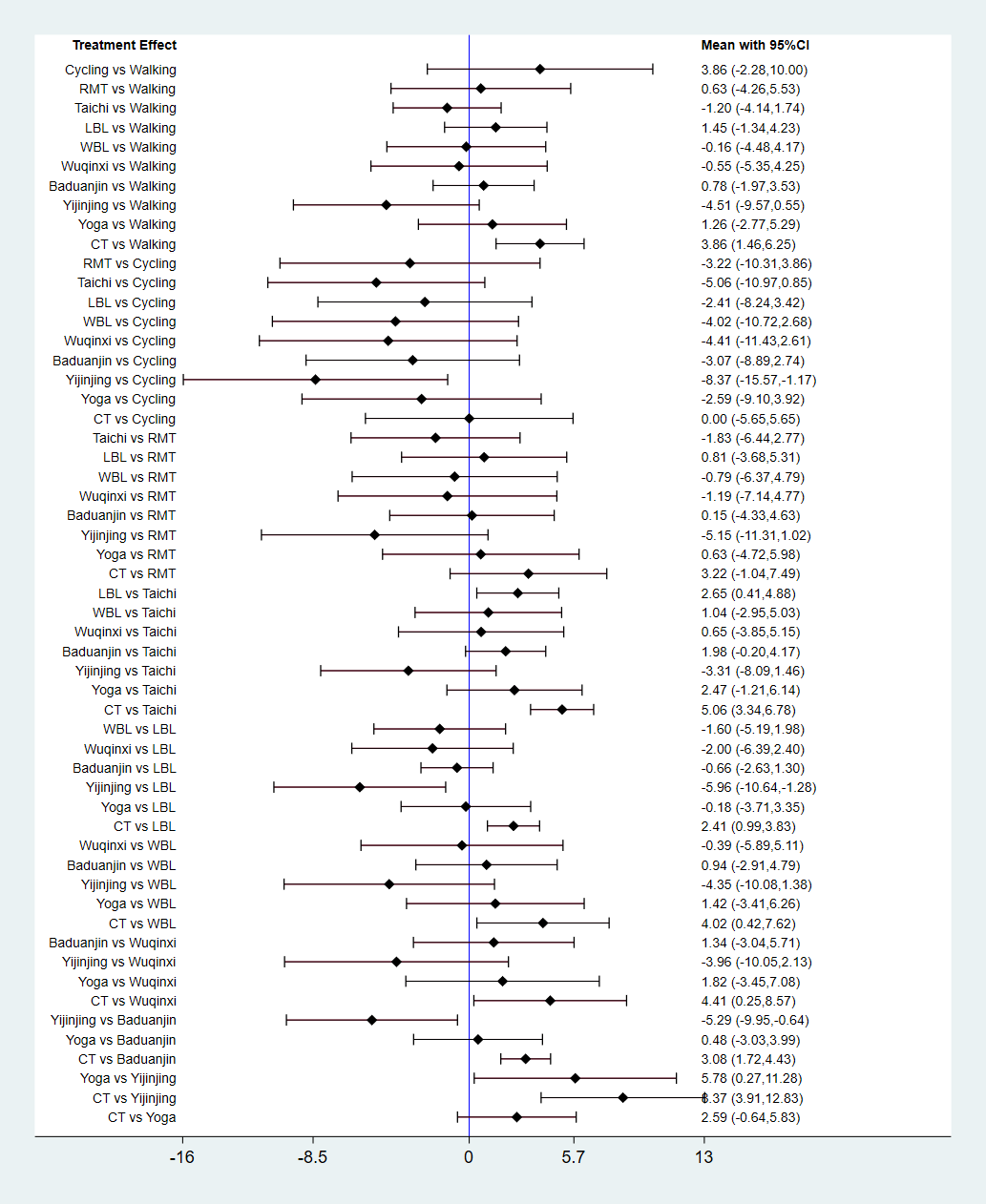
**

**Appendix 9. Cumulative ranking probability curves**

**9.1 Cumulative ranking probability curves FEV_1_%pred**


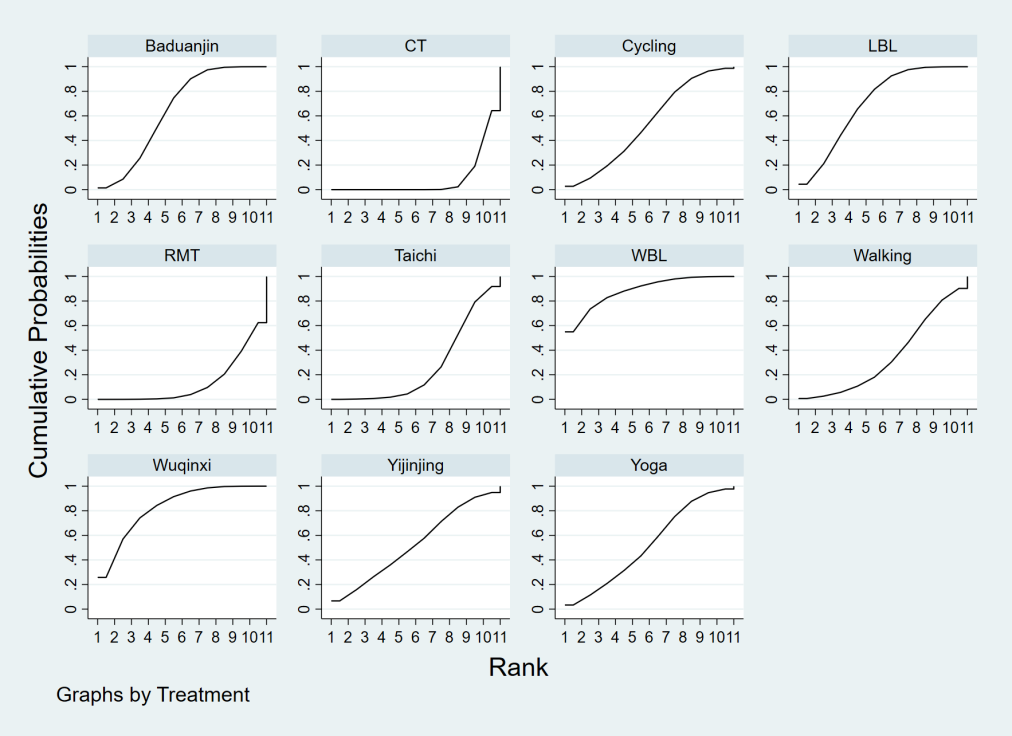


**9.2 Cumulative ranking probability curves CAT**


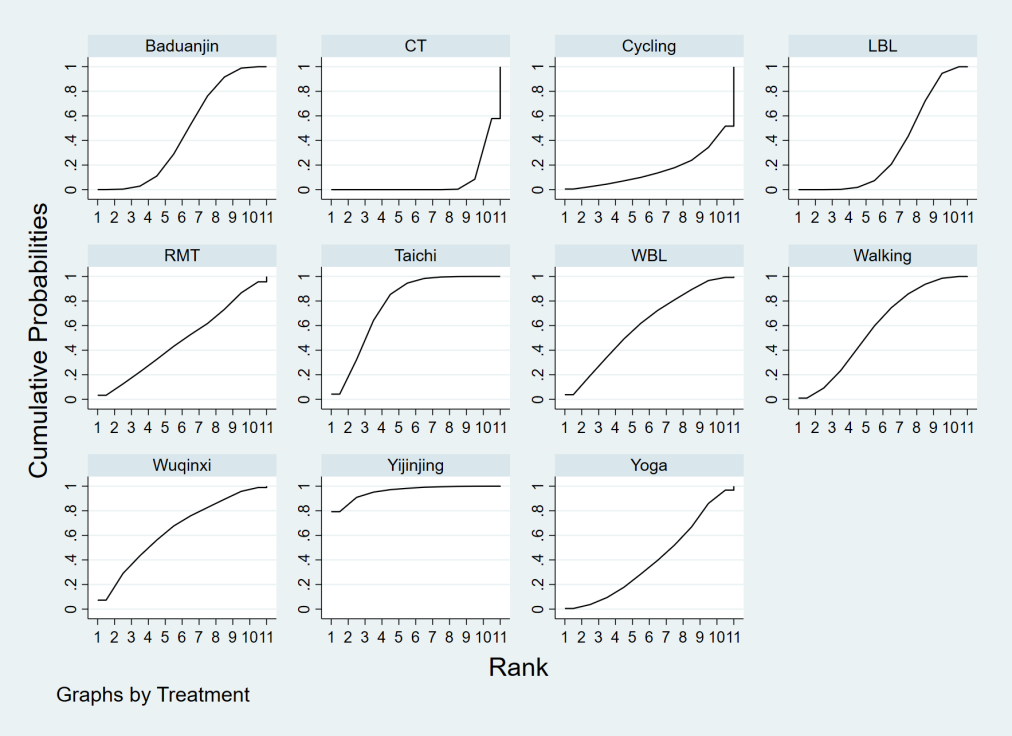


**Appendix 10 Sensitivity analysis**

**10.1 Sensitivity analysis excluding studies with sample size < 30**

**10.1.1 Forest Plot of FEV_1_%pred**

**
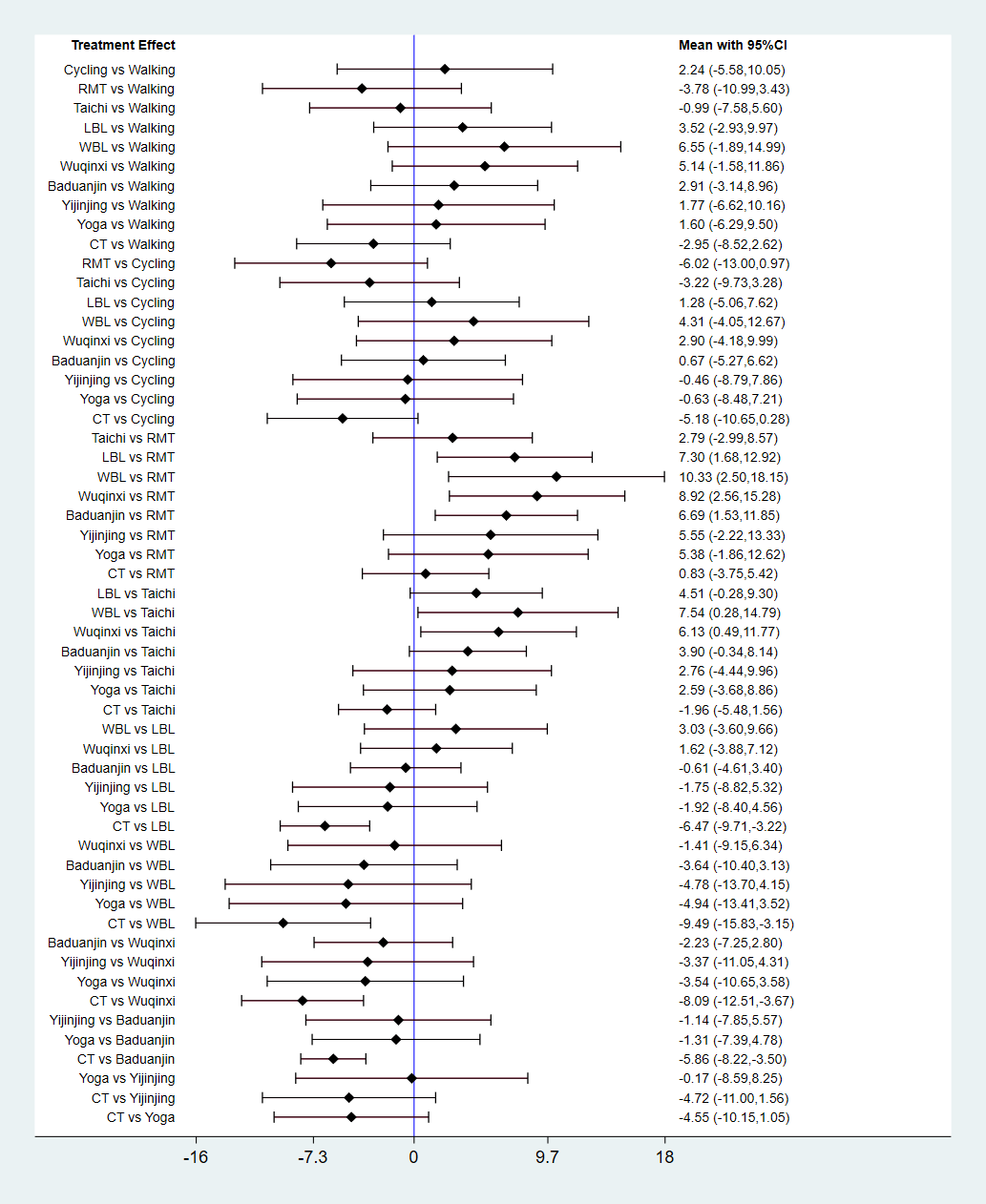
**

**10.1.2 Network meta-analysis forest plots on CAT**

**
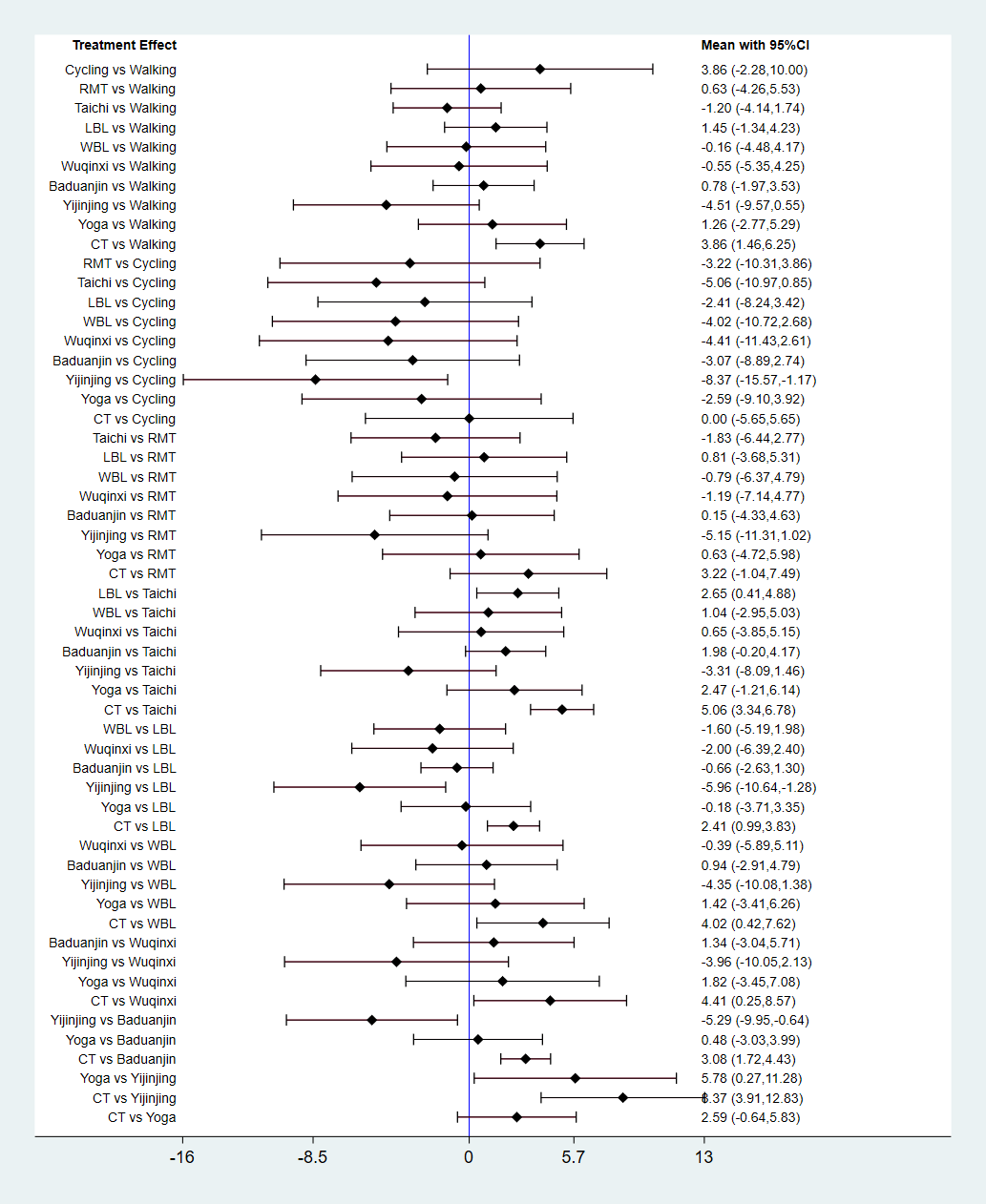
**

**10.1.3 Ranking table of SUCRA values**

| Intervention | FEV_1_%pred | CAT |
| --- | --- | --- |
| Walking | 36.8 | 58.7 |
| Cycling | 57.2 | 16.4 |
| RMT | 9.3 | 49.9 |
| Taichi | 27.9 | 77.9 |
| LBL | 69.6 | 33.7 |
| WBL | 87.8 | 60.3 |
| Wuqinxi | 82.2 | 64.4 |
| Baduanjin | 63.8 | 46.4 |
| Yijinjing | 52.9 | 95.9 |
| Yoga | 51.6 | 39.8 |
| CT | 10.9 | 6.6 |

**10.2 Sensitivity analysis excluding studies with intervention duration < 12 weeks**

**10.2.1 Forest Plot of FEV_1_%pred**


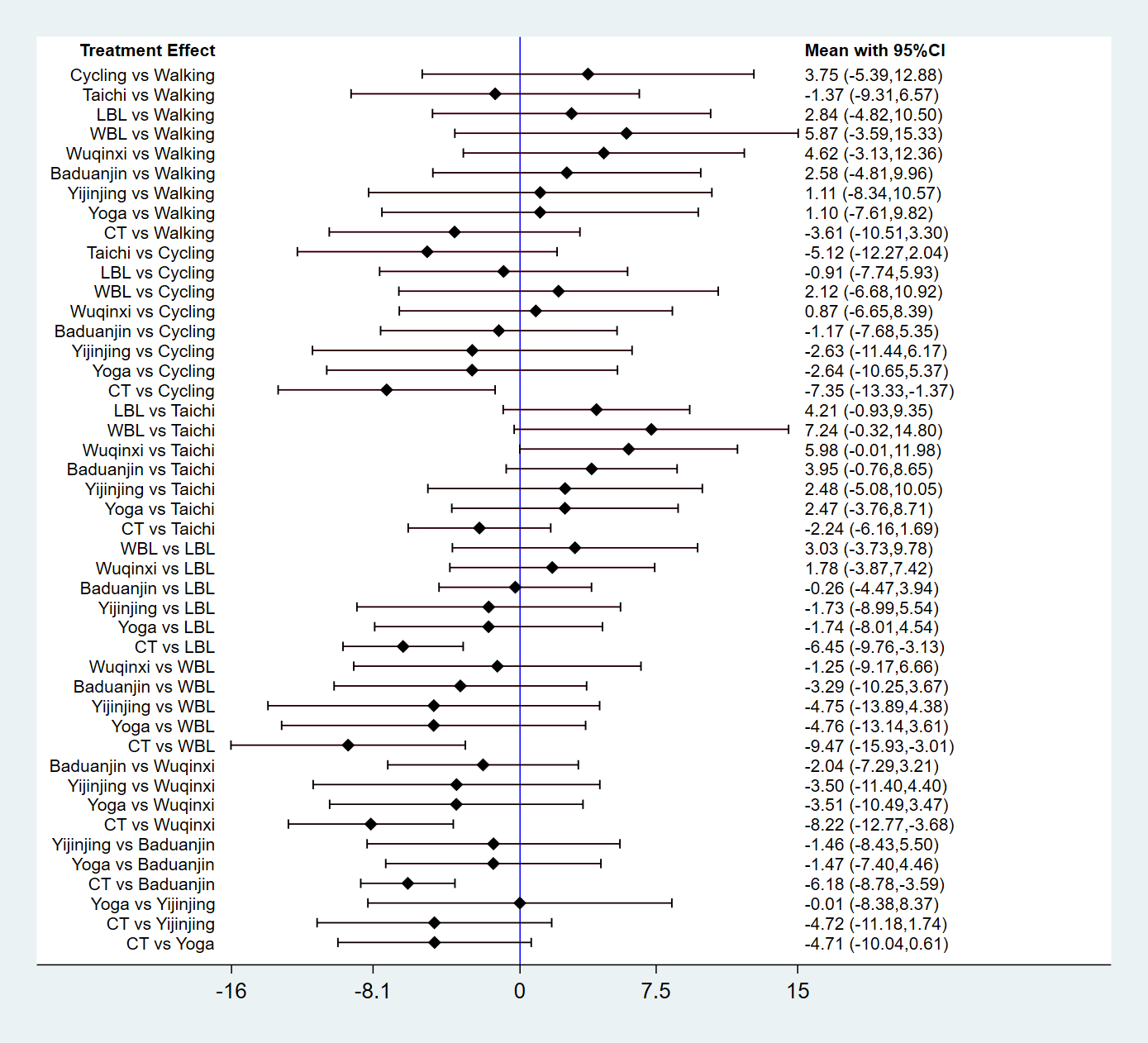


**10.2.2 Network meta-analysis forest plots on CAT**


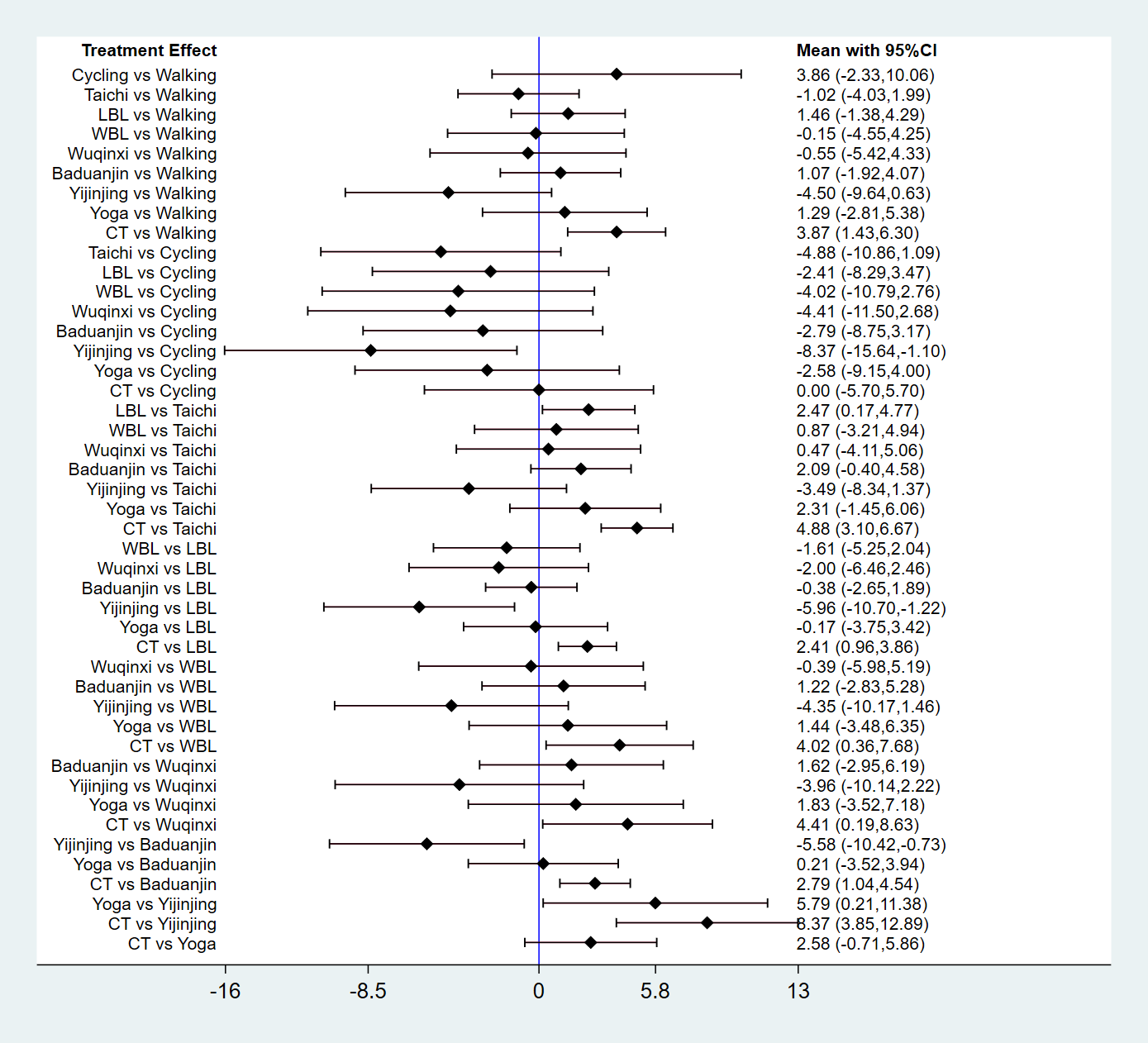


**10.2.3 Ranking table of SUCRA values**

| Intervention | FEV_1_%pred | CAT |
| --- | --- | --- |
| Walking | 36.0 | 59.8 |
| Cycling | 68.3 | 17.2 |
| Taichi | 21.8 | 76.0 |
| LBL | 60.8 | 35.7 |
| WBL | 83.6 | 60.2 |
| Wuqinxi | 76.8 | 65.0 |
| Baduanjin | 58.3 | 42.4 |
| Yijinjing | 45.3 | 96.4 |
| Yoga | 44.5 | 40.6 |
| CT | 4.4 | 6.8 |

**Appendix 11 Egger's test**

| Outcome | Coef. | SE | t | 1. **\| t \|** | 95% CI |
| --- | --- | --- | --- | --- | --- |
| FEV_1_%pred | -0.515 | 0.509 | -1.01 | 0.316 | (-1.529, 0.500) |
| CAT | 0.496 | 0.886 | 0.56 | 0.579 | (-1.305, 2.296) |

# References

[1] Yu J. Effect of 6-minute walking training on dyspnea in patients with COPD. Chin J Rehabil Theory Pract. 2003;(09):52-53.

[2] Li SX, Zhang Y, Ma XJ. Ground walking training can improve dyspnea in patients with stable chronic obstructive pulmonary disease. Dis Monit Control. 2015;9(10):704-705.

[3] Duan YJ, Tang ZH, Chen XB, et al. Effect of 6-minute walking exercise on quality of life in patients with chronic obstructive pulmonary disease. Sichuan Med J. 2016;37(06):623-625. https://doi.org/10.16252/j.cnki.issn1004-0501-2016.06.013.

[4] Jin YL, Yao J, Hou LL. Effect of exercise training on activity tolerance in patients with chronic obstructive pulmonary disease. Shanghai Nurs. 2016;16(06):20-23.

[5] Chen YH, Chen LR, Tsao CC, et al. Effects of a pedometer-based walking program in patients with COPD-a pilot study. Medicina (Kaunas). 2022;58(4):490. https://doi.org/10.3390/medicina58040490.

[6] Cui SL, Jiang WP, Zhu HL, et al. Effects of upper- and lower-limb combined exercise training on cardiopulmonary function in elderly patients with stable chronic obstructive pulmonary disease. Chin J Respir Crit Care Med. 2011;10(02):107-111.

[7] Zhang J, He ZY, Liu Y, et al. Prescription of lower-limb exercise training under pulmonary rehabilitation in patients with stable chronic obstructive pulmonary disease. Chin J Clin Physicians (Electronic Edition). 2012;6(12):3273-3278.

[8] Wu H, Sun XG, Gu WC, et al. Clinical report of submaximal lower-limb exercise on a cycle ergometer for rehabilitation in chronic obstructive pulmonary disease. Chin J Appl Physiol. 2015;31(04):382-384.

[9] Duruturk N, Arıkan H, Ulubay G, et al. A comparison of calisthenic and cycle exercise training in chronic obstructive pulmonary disease patients: a randomized controlled trial. Expert Rev Respir Med. 2016;10(1):99-108. https://doi.org/10.1586/17476348.2015.1126419.

[10] Wang XJ, Wu T, Wang YC. Efficacy of cycle aerobic exercise combined with inhaled medication in patients with chronic obstructive pulmonary disease. Chin J Gerontol. 2018;38(20):4935-4936.

[11] Scherer TA, Spengler CM, Owassapian D, et al. Respiratory muscle endurance training in chronic obstructive pulmonary disease: impact on exercise capacity, dyspnea, and quality of life. Am J Respir Crit Care Med. 2000;162(5):1709-1714. https://doi.org/10.1164/ajrccm.162.5.9912026.

[12] Hill K, Jenkins SC, Philippe DL, et al. High-intensity inspiratory muscle training in COPD. Eur Respir J. 2006;27(6):1119-1128. https://doi.org/10.1183/09031936.06.00105205.

[13] Bavarsad MB, Shariati A, Eidani E, et al. The effect of home-based inspiratory muscle training on exercise capacity, exertional dyspnea and pulmonary function in COPD patients. Iran J Nurs Midwifery Res. 2015;20(5):613-618. https://doi.org/10.4103/1735-9066.164588.

[14] Wu W, Guan L, Zhang X, et al. Effects of two types of equal-intensity inspiratory muscle training in stable patients with chronic obstructive pulmonary disease: a randomised controlled trial. Respir Med. 2017;132:84-91. https://doi.org/10.1016/j.rmed.2017.10.001.

[15] Leelarungrayub J, Pinkaew D, Puntumetakul R, et al. Effects of a simple prototype respiratory muscle trainer on respiratory muscle strength, quality of life and dyspnea, and oxidative stress in COPD patients: a preliminary study. Int J Chron Obstruct Pulmon Dis. 2017;12:1415-1425. https://doi.org/10.2147/COPD.S131062.

[16] Xu W, Li R, Guan L, et al. Combination of inspiratory and expiratory muscle training in same respiratory cycle versus different cycles in COPD patients: a randomized trial. Respir Res. 2018;19(1):225. https://doi.org/10.1186/s12931-018-0917-6.

[17] Saka S, Gurses HN, Bayram M. Effect of inspiratory muscle training on dyspnea-related kinesiophobia in chronic obstructive pulmonary disease: a randomized controlled trial. Complement Ther Clin Pract. 2021;44:101418. https://doi.org/10.1016/j.ctcp.2021.101418.

[18] Majewska-Pulsakowska M, Wytrychowski K, Rożek-Piechura K. The role of inspiratory muscle training in the process of rehabilitation of patients with chronic obstructive pulmonary disease. Adv Exp Med Biol. 2016;885:47-51. https://doi.org/10.1007/5584_2015_194.

[19] Du ST, Ding LM, Wang CX, et al. Effect of Tai Chi exercise on exercise endurance and pulmonary function in patients with chronic obstructive pulmonary disease. Chin J Rehabil Med. 2013;28(04):374-376.

[20] Zhang XC, Cai YL, Zhang W, et al. Effects of 24-form Tai Chi and respiratory function training combined with western medicine in patients with stable chronic obstructive pulmonary disease. J Tradit Chin Med. 2014;55(22):1937-1941.

[21] Li SX, Li AJ, Sun J, et al. Effect of Tai Chi rehabilitation on patients with chronic obstructive pulmonary disease. J Binzhou Med Univ. 2016;39(04):275-277+284.

[22] Pan Y, Wang ZX, Min J, et al. Evaluation of simplified 24-form Tai Chi in pulmonary rehabilitation for patients with stable chronic obstructive pulmonary disease. Chin J Rehabil Med. 2018;33(06):681-686.

[23] Zhu S, Shi K, Yan J, et al. A modified 6-form Tai Chi for patients with COPD. Complement Ther Med. 2018;39:36-42. https://doi.org/10.1016/j.ctim.2018.05.007.

[24] Liu DH, Zhang Y. Effect of Tai Chi exercise on pulmonary function in middle-aged patients with stable chronic obstructive pulmonary disease. Chin Community Doctors. 2019;35(16):154+157.

[25] Wang L, Wu K, Chen X, et al. The effects of Tai Chi on lung function, exercise capacity and health related quality of life for patients with chronic obstructive pulmonary disease: a pilot study. Heart Lung Circ. 2019;28(8):1206-1212. https://doi.org/10.1016/j.hlc.2018.05.204.

[26] Peng HY, Wang P, Wang YD, et al. Effect of six-form Tai Chi rehabilitation exercise on pulmonary rehabilitation in patients with stable chronic obstructive pulmonary disease. Shanghai Med Pharm J. 2020;41(22):58-62.

[27] Ni L. Observation on the efficacy of yoga and Tai Chi in the remission stage of COPD. World Latest Med Inf. 2017;17(92):182+184.

[28] Chen JX, Zhang WX, Zheng GH, et al. Application of Liuzijue breathing exercise in pulmonary rehabilitation of patients with stable COPD. J Fujian Coll Tradit Chin Med. 2008;18(06):3-4.

[29] Lan Y, Han X, Wang YY, et al. Effects of tiotropium bromide combined with Liuzijue exercise on quality of life and pulmonary function in patients with stable COPD. World J Integr Tradit West Med. 2016;11(10):1369-1371+1395.

[30] Shen Q. Community intervention study of Liuzijue health exercise in elderly patients with stable COPD (grade I). Clin J Chin Med. 2017;9(23):23-25.

[31] Li P, Liu J, Lu Y, et al. Effects of long-term home-based Liuzijue exercise combined with clinical guidance in elderly patients with chronic obstructive pulmonary disease. Clin Interv Aging. 2018;13:1391-1399. https://doi.org/10.2147/CIA.S169671.

[32] Wu W, Liu X, Li P, et al. Effect of Liuzijue exercise combined with elastic band resistance exercise on patients with COPD: a randomized controlled trial. Evid Based Complement Alternat Med. 2018;2018:2361962. https://doi.org/10.1155/2018/2361962.

[33] Wu W, Liu X, Liu J, et al. Effectiveness of water-based Liuzijue exercise on respiratory muscle strength and peripheral skeletal muscle function in patients with COPD. Int J Chron Obstruct Pulmon Dis. 2018;13:1713-1726. https://doi.org/10.2147/COPD.S165593.

[34] Ji SQ, Luo GW, Shi KJ, et al. Clinical study of Liuzijue intervention in patients with stable chronic obstructive pulmonary disease. West J Tradit Chin Med. 2019;32(04):111-114.

[35] Shi XL, Ji SQ, Jiang FY, et al. Study on the efficacy of Liuzijue in the treatment of stable chronic obstructive pulmonary disease. Liaoning J Tradit Chin Med. 2020;47(09):103-106.

[36] Deng LJ, Chen JX, Chen YN, et al. Effects of Liuzijue on elderly patients with chronic obstructive pulmonary disease of lung qi deficiency syndrome. Med Innov China. 2020;17(36):112-117.

[37] Lu F, Wang SC. A randomized controlled multicenter clinical study of Liuzijue breathing exercise in the treatment of stable COPD. Fujian J Tradit Chin Med. 2021;52(06):1-3.

[38] Hu JB, Wei L, Tu HB, et al. Rehabilitation effect of water-based Liuzijue on patients with stable chronic obstructive pulmonary disease. J Clin Med Pract. 2021;25(10):45-47+61.

[39] Yan Y. Evaluation of the efficacy of traditional Chinese Liuzijue training combined with conventional western medicine in chronic obstructive pulmonary disease. West J Tradit Chin Med. 2021;34(05):126-129.

[40] Fang Y, Fang Q, Wang SQ, et al. Effects of simplified Liuzijue combined with external diaphragmatic pacing on SGRQ score, 6MWD and quality of life in patients with stable COPD. Guangdong Med J. 2022;43(12):1551-1556.

[41] Chen F. Effect of enhanced Liuzijue on quality of life in patients with stable chronic obstructive pulmonary disease in plateau areas. Plateau Med J. 2024;34(01):40-43.

[42] Xu XG, Zhu XX, Sun GL, et al. Efficacy evaluation of Liuzijue breathing exercise in elderly patients with stable chronic obstructive pulmonary disease. J Jilin Med Univ. 2026:1-7.

[43] Cao Q, Wang ZW, Ji SQ, et al. Effect of water-based Liuzijue on pulmonary function in patients with stable chronic obstructive pulmonary disease. Tradit Chin Med J. 2022;21(06):40-42+60.

[44] Zhu Y, Li N, Jin HZ. Effect of early intervention with Wuqinxi on patients with stable chronic obstructive pulmonary disease. J Liaoning Univ Tradit Chin Med. 2010;12(06):107-110.

[45] Wei SS, Cheng YF, He R. Effect of traditional Hua Tuo Wuqinxi on pulmonary function in patients with stable chronic obstructive pulmonary disease. Clin J Tradit Chin Med. 2015;27(06):793-795.

[46] Cheng YF, Wei SS, He R. Observation on the clinical efficacy of traditional Hua Tuo Wuqinxi in patients with stable chronic obstructive pulmonary disease. Clin J Tradit Chin Med. 2015;27(05):683-685.

[47] Zhao QL. Clinical study of Wuqinxi in the prevention and treatment of community patients with stable chronic obstructive pulmonary disease. Acta Chin Med. 2015;30(06):801-803.

[48] Gao YF, Ou YY, Chen MY. Effect of Wuqinxi exercise on pulmonary function and exercise tolerance in patients with chronic obstructive pulmonary disease during the post-discharge transition period. J Clin Pathol Res. 2017;37(05):975-980.

[49] Liu FY, Tan PH. Application of community Wuqinxi exercise in patients with stable chronic obstructive pulmonary disease. J Henan Med Coll. 2020;32(05):522-524.

[50] Feng YC, Pan HS, Wen X, et al. Observation on the therapeutic effect of Baduanjin exercise in elderly patients with stable chronic obstructive pulmonary disease. J New Chin Med. 2009;41(08):36-37.

[51] Liu SR, Chen YF. Clinical study on the effect of health qigong Baduanjin on improving 6-minute walking distance in patients with stable chronic obstructive pulmonary disease. Sichuan Med J. 2013;34(08):1090-1092.

[52] Zhu ZG, Chen Y. Effect of seated Baduanjin exercise on pulmonary function in patients with COPD. World J Integr Tradit West Med. 2014;9(08):846-848.

[53] Chen JX, Deng YF, Chen Q, et al. Effects of the third form of Baduanjin on quality of life and exercise endurance in patients with chronic obstructive pulmonary disease of lung-spleen qi deficiency syndrome. Rehabil Med J. 2015;25(03):13-17.

[54] Deng YF, Chen JX. Effect of the single-lift form of Baduanjin on rehabilitation outcomes in patients with chronic obstructive pulmonary disease. Chin J Nurs. 2015;50(12):1458-1463.

[55] Liang XL. Effect of rehabilitation exercise with the single-lift form of Baduanjin on patients with chronic obstructive pulmonary disease. Nurs Pract Res. 2016;13(17):156-157.

[56] Pan MY, Luo JH, Yang SC. Observation on the efficacy of standing-posture Baduanjin rehabilitation exercise in patients with stable chronic obstructive pulmonary disease. J Chengdu Univ Tradit Chin Med. 2016;39(03):49-52.

[57] Guo JC, Gao YF, Xie HX, et al. Effect of Baduanjin exercise on rehabilitation outcomes in patients with stable chronic obstructive pulmonary disease. Qilu Nurs J. 2016;22(07):97-98.

[58] Guo JC. Efficacy analysis of traditional health exercise Baduanjin in promoting pulmonary rehabilitation in patients with stable COPD. J Shandong Med Coll. 2016;38(03):171-174.

[59] Huang BJ, Yao QP, Zhu YM. Observation on the efficacy of health qigong Baduanjin as adjuvant therapy for stable chronic obstructive pulmonary disease of lung-spleen deficiency syndrome. Hubei J Tradit Chin Med. 2017;39(01):4-6.

[60] Zhang LX, Wang T, Shi L, et al. Effect of Baduanjin “regulating zong qi” on nutritional status and cardiopulmonary rehabilitation assessment in patients with chronic obstructive pulmonary disease. J Changchun Univ Tradit Chin Med. 2017;33(06):954-956. https://doi.org/10.13463/j.cnki.cczyy.2017.06.031.

[61] Zhu ZG, Fang S, Liang BH, et al. Effect of seated Baduanjin exercise on airway inflammatory response in patients with severe stable COPD. J Nurs Adm. 2017;17(01):55-57.

[62] Wang L, Fang L. Effect of practicing Baduanjin on pulmonary function in patients with stable chronic obstructive pulmonary disease. Guid J Tradit Chin Med Pharm. 2018;24(03):86-87+91.

[63] Dong LJ, Liu B. Effects of Shaolin Baduanjin on BODE index and quality of life in patients with stable chronic obstructive pulmonary disease. Clin J Tradit Chin Med. 2018;30(08):1465-1467.

[64] Zhang TT, Ma XQ, Chen RH, et al. Evaluation of the effects of regular Baduanjin exercise on pulmonary function, fatigue, and activity tolerance in patients with stable COPD. World J Integr Tradit West Med. 2019;14(03):415-418.

[65] Yu YY. Evaluation of the efficacy of health qigong Baduanjin in pulmonary rehabilitation of patients with stable chronic obstructive pulmonary disease. World Latest Med Inf. 2019;19(84):210-211.

[66] Deng W, Yang LL, Dong HZ, et al. Evaluation of the efficacy of Deng’s health-preserving Baduanjin in pulmonary rehabilitation of chronic obstructive pulmonary disease. Massage Rehabil Med. 2020;11(24):41-44.

[67] Liu GL. Effects of Baduanjin on patients with stable chronic obstructive pulmonary disease. J Shandong Med Coll. 2021;43(05):389-391.

[68] Feng LY, Zhang WJ, Huang JJ, et al. Effects of Baduanjin training on lower-limb muscle strength and quality of life in patients with chronic obstructive pulmonary disease. Shenzhen J Integr Tradit Chin West Med. 2024;34(12):129-131.

[69] Chen Y, Zhang P, Dong Z, et al. Effect of Baduanjin exercise on health and functional status in patients with chronic obstructive pulmonary disease: a community-based, cluster-randomized controlled trial. NPJ Prim Care Respir Med. 2024;34(1):43. https://doi.org/10.1038/s41533-024-00400-y.

[70] Chen X, Fu C, Wang X, et al. Combined effect of 12 weeks Baduanjin and tri-ball respiratory training as a home-based pulmonary rehabilitation in subjects with moderate chronic obstructive pulmonary disease: a multi-center randomized controlled trial. Rehabil Nurs. 2025;50(2):78-87. https://doi.org/10.1097/RNJ.0000000000000493.

[71] Zhang TT, Zhu TF, Chen RH, et al. Application of respiratory Baduanjin in patients with stable chronic obstructive pulmonary disease and its effects on blood gas indices and CAT and MCFS scores. Liaoning J Tradit Chin Med. 2025;52(08):120-123.

[72] Zhang M, Xu GH, Li F, et al. Health qigong Yijinjing promotes rehabilitation in patients with stable chronic obstructive pulmonary disease. Chin J Sports Med. 2016;35(04):339-343.

[73] Zhang M, Xv G, Luo C, et al. Qigong Yi Jinjing promotes pulmonary function, physical activity, quality of life and emotion regulation self-efficacy in patients with chronic obstructive pulmonary disease: a pilot study. J Altern Complement Med. 2016;22(10):810-817. https://doi.org/10.1089/acm.2015.0224.

[74] Donesky-Cuenco D, Nguyen HQ, Paul S, et al. Yoga therapy decreases dyspnea-related distress and improves functional performance in people with chronic obstructive pulmonary disease: a pilot study. J Altern Complement Med. 2009;15(3):225-234. https://doi.org/10.1089/acm.2008.0389.

[75] Gupta A, Gupta R, Sood S, et al. Pranayam for treatment of chronic obstructive pulmonary disease: results from a randomized, controlled trial. Integr Med (Encinitas). 2014;13(1):26-31.

[76] Kaminsky DA, Guntupalli KK, Lippmann J, et al. Effect of yoga breathing (pranayama) on exercise tolerance in patients with chronic obstructive pulmonary disease: a randomized, controlled trial. J Altern Complement Med. 2017;23(9):696-704. https://doi.org/10.1089/acm.2017.0102.

[77] Yudhawati R, Rasjid Hs M. Effect of yoga on FEV1, 6-minute walk distance (6-MWD) and quality of life in patients with COPD group B. Adv Respir Med. 2019;87(5):261-268. https://doi.org/10.5603/ARM.2019.0047.
